# TRIM39 deficiency inhibits tumor progression and autophagic flux in colorectal cancer via suppressing the activity of Rab7

**DOI:** 10.1038/s41419-021-03670-3

**Published:** 2021-04-12

**Authors:** Jia Hu, Xueliang Ding, Shaobo Tian, Yanan Chu, Zhibo Liu, Yuqin Li, Xiaoqiong Li, Guobin Wang, Lin Wang, Zheng Wang

**Affiliations:** 1 Research Center for Tissue Engineering and Regenerative Medicine, Union Hospital, Tongji Medical College, Huazhong University of Science and Technology, 430022 Wuhan, China; 2 Department of Gastrointestinal Surgery, Union Hospital, Tongji Medical College, Huazhong University of Science and Technology, 430022 Wuhan, China; 3 Department of Clinical Laboratory, Union Hospital, Tongji Medical College, Huazhong University of Science and Technology, 430022 Wuhan, China

**Keywords:** Colorectal cancer, Oncogenes

## Abstract

The biological function of TRIM39, a member of TRIM family, remains largely unexplored in cancer, especially in colorectal cancer (CRC). In this study, we show that TRIM39 is upregulated in tumor tissues compared to adjacent normal tissues and associated with poor prognosis in CRC. Functional studies demonstrate that TRIM39 deficiency restrains CRC progression in vitro and in vivo. Our results further find that TRIM39 is a positive regulator of autophagosome–lysosome fusion. Mechanistically, TRIM39 interacts with Rab7 and promotes its activity via inhibiting its ubiquitination at lysine 191 residue. Depletion of TRIM39 inhibits CRC progression and autophagic flux in a Rab7 activity-dependent manner. Moreover, TRIM39 deficiency suppresses CRC progression through inhibiting autophagic degradation of p53. Thus, our findings uncover the roles as well as the relevant mechanisms of TRIM39 in CRC and establish a functional relationship between autophagy and CRC progression, which may provide promising approaches for the treatment of CRC.

## Introduction

Colorectal cancer (CRC), the third most common malignant tumor worldwide in 2018, evolves with a series of genetic alterations and pathway dysregulation^1–3^. Among these mechanisms, dysregulation of autophagy is an important event for initiation and development of CRC^4^. Autophagy is a catabolic process characterized by a double-membrane vesicle called autophagosome that packages aggregated components and damaged organelles for degradation^5^. Autophagy process can be artificially divided into three phases: autophagosome induction and nucleation, autophagosome elongation, autophagosome maturation and degradation. It is generally believed that inhibiting autophagosome maturation and degradation can suppress CRC^6,7^. Chloroquine (CQ) and its derivatives, commonly used autophagosome–lysosome fusion inhibitors via suppressing lysosomal acidification, have been reported to inhibit growth and reduce chemotherapy resistance in CRC^8–10^. However, sorting nexin 10, which can promote autophagosome–lysosome fusion by binding with ATG12–ATG5 conjugate and lysosome-associated membrane protein 1 (LAMP1) to mediate autophagic degradation of SRC, suppressed initiation and progression of CRC^11^. These studies suggest that targeting different molecules and pathways in autophagosome maturation and degradation process may induce distinct effects on CRC.

Tripartite motif family (TRIM) is a large family of proteins that usually have E3 ubiquitin ligase activity^12^. Some members of TRIM have been reported to involve in regulation of both autophagy and cancer^12,13^. TRIM59 promoted breast cancer invasion by suppressing p62-selective autophagic degradation of programmed cell death protein 10^14^. TRIM65 knockdown attenuated autophagy and cisplatin resistance in non-small cell lung carcinoma^15^. Tripartite motif 39 (TRIM39), also known as Ring finger protein 23, participated in the regulation of immune responses, cell cycle, and apoptosis^16–18^. TRIM39 can negatively regulate nuclear factor-κB signaling through stabilization of Cactin^16^. TRIM39 also modulates the stability of p53 and its downstream target p21 to affect cell cycle and apoptosis^17,19^. However, the functional roles of TRIM39 in autophagy and CRC are still unexplored.

Here we found that TRIM39 was upregulated in CRC tumor tissues and associated with poor clinical outcomes of CRC patients. In vitro and in vivo results demonstrated that TRIM39 knockdown suppressed the growth and metastasis of CRC. TRIM39 deficiency also inhibited autophagic flux in CRC. Mechanistically, Rab7 was identified as a TRIM39-interacting protein and this interaction could enhance Rab7 activity. We further found that TRIM39 promoted Rab7 activity via inhibiting its ubiquitination at lysine 191 residue. TRIM39 deficiency suppressed CRC progression and autophagic flux in a Rab7 activity-dependent manner. Moreover, TRIM39 knockdown dampened CRC progression via inhibiting autophagic degradation of p53. This work may provide new approaches and targets for CRC treatment.

## Materials and methods

### Antibodies and reagents

Antibodies used in this study were as follows: anti-Rab7 (Santa Cruz Biotechnology, sc-376362), anti-Rab7 (Proteintech, 55469-1-AP), anti-p53 (Santa Cruz Biotechnology, sc-126), anti-SQSTM1/p62 (Proteintech, 18420-1-AP), anti-LC3B (MBL, M186-3), anti-RILP (Proteintech, 13574-1-AP), anti-GDI2 (Proteintech, 10116-1-AP), anti-LAMP1 (Proteintech, 55273-1-AP), anti-MON1A (Proteintech, 23772-1-AP), anti-CD63 (Proteintech, 25682-1-AP), anti-CTSB (Proteintech, 12216-1-AP), anti-CTSD (Proteintech, 21327-1-AP), anti-EEA1 (CUSABIO, CSB-PA007405LA01HU), Anti-EEA1 mAb-Alexa Fluor^®^ 488 (MBL, M176-A48), anti-GAPDH (Proteintech, 60004-1-Ig), anti-MYC (Santa Cruz Biotechnology, sc-40), anti-FLAG (Biolegend, 637301), anti-GFP (Proteintech, 66002-1-Ig), anti-HA (Proteintech, 51064-2-AP), anti-GST (Proteintech, 10000-0-AP). Other reagents used in this study were: Hoechst 33342 (Sigma Aldrich, 14533), CQ (Sigma-Aldrich, C6628), Lyso-Tracker Red (Beyotime, C1046), and Lipofectamine® 2000 (Invitrogen, 11668019).

### Cell culture, transfections, and treatments

HCT116, LoVo, DLD-1, HT29, SW48, SW480, Caco2, and HEK293T cells were purchased from American Type Culture Collection and cultured in Dulbecco’s Modified Eagle’s Medium (DMEM; Hyclone, SH30022.01) supplemented with 10% fetal bovine serum (FBS). Cells were transfected using Lipofectamine 2000 according to the manufacturer’s instruction. Autophagy was induced by nutrient deprivation through incubation in Earle’s balanced salt solution. The inhibition of autophagic flux was achieved by treating cells with 50 nM CQ, which could block the fusion of autophagosomes and lysosomes.

### Plasmid construction and small interfering RNAs (siRNAs)

Human TRIM39 cDNA was amplified from cDNA library of HEK293T cells and cloned into pcDNA3.1/Myc-His (Invitrogen), pFLAG-CMV, or pEGFP-C1 vectors. Human Rab7 cDNA was cloned into pcDNA3.1/Myc-His, pEGFP-C1, or pLenti-neo vectors. Oligonucleotide-specific short hairpin RNAs (shRNAs) targeting human TRIM39 or Rab7 were synthesized and then cloned into pLKO.1-puro or pLKO.1-neo vectors. All plasmids were confirmed by DNA sequencing. The shRNA sequences in this study are listed in Table S1. siRNA targeting TRIM39 (siTRIM39) and non-targeting siRNA negative control (siNC) were purchased from RIBOBIO. The sequence information was listed in Table S1.

### Lentivirus production and infection

HEK293T cells were co-transfected with pLKO.1-shRNA, psPAX2 packaging, and pMD2.G enveloped plasmids according to the manufacturer’s instructions. The supernatant containing lentiviral particles was harvested at 48 h post transfection. CRC cell lines were infected with filtered lentivirus in the presence of polybrene (8 μg/mL) and then selected by puromycin (1 μg/mL) or neomycin (300 μg/mL) for 1 week. The knockdown efficiency was identified by western blot or reverse transcription polymerase chain reaction (RT-PCR).

### Colony-formation assay and soft agar colony-formation assay

For colony-formation assay, HCT116 cells or LoVo cells (500 cells/well) were seeded in 12-well plates and cultured for 2 weeks. Colonies were fixed and stained with crystal violet. The number of colonies was counted.

For soft agar colony-formation assay, HCT116 cells (1500 cells/well) or LoVo cells (2000 cells/well) were suspended with 0.4% w/v agar and then the cell mixture was added on the top of a 0.8% w/v base agar layer supplemented with DMEM containing 10% FBS in 12-well plates. After 12 days of incubation, colonies were fixed and stained with 0.05% crystal violet. The number of colonies was counted.

### Cell migration and invasion assay

The migratory and invasive ability of cells were evaluated using 24-well Boyden chambers (Corning Inc., 3422). For migration assay, cells were resuspended in serum-free DMEM and seeded in the upper chamber (0.5 × 10^5^ LoVo cells or 1 × 10^5^ HCT116 cells). The lower chamber was added with 500 μL DMEM medium containing 10% FBS. After 12 h (LoVo) or 36 h (HCT116), cells were fixed and stained with crystal violet.

For invasion assay, the upper chamber was pre-coated with matrigel matrix (BD Science). In all, 2.5 × 10^5^ LoVo cells or HCT116 cells were added to the Matrigel-coated upper chamber and allowed to invade 36 h for LoVo cells and 72 h for HCT116 cells. Invaded cells were fixed and stained with crystal violet. The representative images were taken by microscope.

### PolyQ degradation assay

Cells were co-transfected with the indicated plasmids and polyQ80-luciferase or polyQ19-luciferase plasmids using Lipo2000. After 48 h, the firefly luciferase activity was measured with the Dual-Luciferase® Reporter Assay System according to the manufacturer’s protocol. The ratio of polyQ80-luciferase/polyQ19-luciferase luminescence signal values was used to reflect the autophagic flux as described previously^20^.

### LysoTracker red staining assay

Cells were stained with 50 nM LysoTracker red for 30 min at 37 °C, then collected and washed with phosphate-buffered saline (PBS). The average fluorescence intensity of cells was detected by flow cytometer (BD LSRFortessa X-20, USA).

### Western blot and immunoprecipitation analysis

Total protein was extracted with RIPA lysis buffer (Beyotime Institute of Biotechnology) containing protease inhibitors. The protein concentration was determined with the BCA Kit (Thermo Scientific). The cell lysate was separated by 12% sodium dodecyl sulfate–polyacrylamide gel electrophoresis and transferred onto nitrocellulose membranes (Bio-Rad, Richmond, CA). After blocking with 5% skimmed milk for 1 h, membranes were incubated with the appropriate primary antibody. The signal from horseradish peroxidase (HRP)-coupled secondary antibodies was detected by the chemiluminescence system (UVP, San Gabriel, CA).

For immunoprecipitation assay, cells were washed with PBS for 3 times, collected, and lysed in immunoprecipitation lysis buffer (Beyotime Institute of Biotechnology) containing protease inhibitors. Cell extracts were incubated with the indicated antibodies overnight at 4 °C. Then protein A/G plus-agarose beads (Santa Cruz, CA, USA) were incubated for another 2 h at 4 °C. The beads were washed three times, boiled at 98 °C for 5 min in protein loading buffer and then subjected to western blot analysis.

### GST-RILP^RBD^ pull down assay

The GST-RILP^RBD^ pull down assay was performed as previously described^21^. Briefly, GST-RILP^RBD^ was expressed in *Escherichia coli* strain BL21 and purified. Equal amount of protein solution was incubated with glutathione-Sepharose 4B beads (GE Healthcare) at 4 °C for 3 h. The beads were washed 5 times with washing buffer, then incubated with whole-cell lysates extracted from transfected cells at 4 °C for 4 h. After washing for 5 times, the beads were boiled in protein loading buffer and subjected to western blot analysis.

### RNA extraction and semi-quantitative RT-PCR

Total RNA was extracted using RNAiso Plus Reagent (Takara, 9109) according to the manufacturer’s protocol. cDNA was synthesized using the M-MLV reverse transcriptase (Vazyme, CHN). PCR reactions were conducted using the 2 × Taq Plus Master Mix (Vazyme, CHN) and set at an initial 95 °C for 3 min and then 22 (glyceraldehyde 3-phosphate dehydrogenase (GAPDH)) and 30 (TRIM39) cycles of 95 °C for 30 s, 56 °C for 30 s, 72 °C for 30 s, and a final extension at 72 °C for 5 min. The products were separated by agarose gel electrophoresis and visualized by ChemiDoc MP System (Bio-Rad, USA). GAPDH was used as an internal control. The primers used in the study are shown in Table S2.

### Immunofluorescence, fluorescence, and confocal microscopy

LoVo or HCT116 cells were seeded in confocal dishes and treated as indicated. Then cells were fixed with 4% paraformaldehyde, permeabilized with 0.2% Triton X-100 (Sigma-Aldrich), blocked with 5% bovine serum albumin overnight, followed by staining with the indicated antibody. Hoechst 33342 was used to stain nuclei. The morphology of the cells was observed and photographed with a Nikon Confocal Microscope.

### Transmission electron microscopy (TEM)

HCT116 cells were fixed with 2.5% glutaraldehyde (pH 7.4) for 2 h at 4 °C and followed by 1.5 h in 1% OsO_4_ (pH 7.4) at room temperature. Then cells were dehydrated in an ascending ethanol series. The specimens were embedded into ultracut (Leica UC7) and sectioned to 60–80 nm. Ultrathin sections were double stained with uranyl acetate and lead citrate and observed with an electron microscope (FEI Tecnai G20, USA).

### Animal studies

Six-week-old male BALB/c nude mice were used for in vivo experiments. In all, 2 × 10^6^ indicated HCT116 cells were subcutaneously injected in the flank of the nude mice. Tumor volume was measured every 2 days and calculated as (length × width^2^)/2. Twenty-one days after injection, the tumors were harvested, weighed, and photographed.

### Immunohistochemistry staining

Xenograft tumor tissues were fixed in 4% formaldehyde solution, embedded with paraffin, and sectioned. The sections were rehydrated and placed into a microwave for antigen retrieval. After being washed with PBS, the slides were incubated with anti-p62 (1:100) overnight, followed with a HRP-conjugated secondary antibody for half an hour, stained with 3,3′-diaminobenzidine and finally counterstained with hematoxylin.

### Statistical analysis

Statistical analysis was performed using GraphPad Prism 6 and shown as mean ± standard deviation (SD) or mean ± standard error of mean (SEM). Survival curves were obtained by using the Kaplan–Meier method and compared according to log-rank test. Other comparisons were conducted by Student’s *t* test and one-way or two-way analysis of variance according to the number of groups. *P* < 0.05 was considered statistically significant.

## Results

### TRIM39 knockdown inhibits tumor progression in CRC

**Fig. 1:**
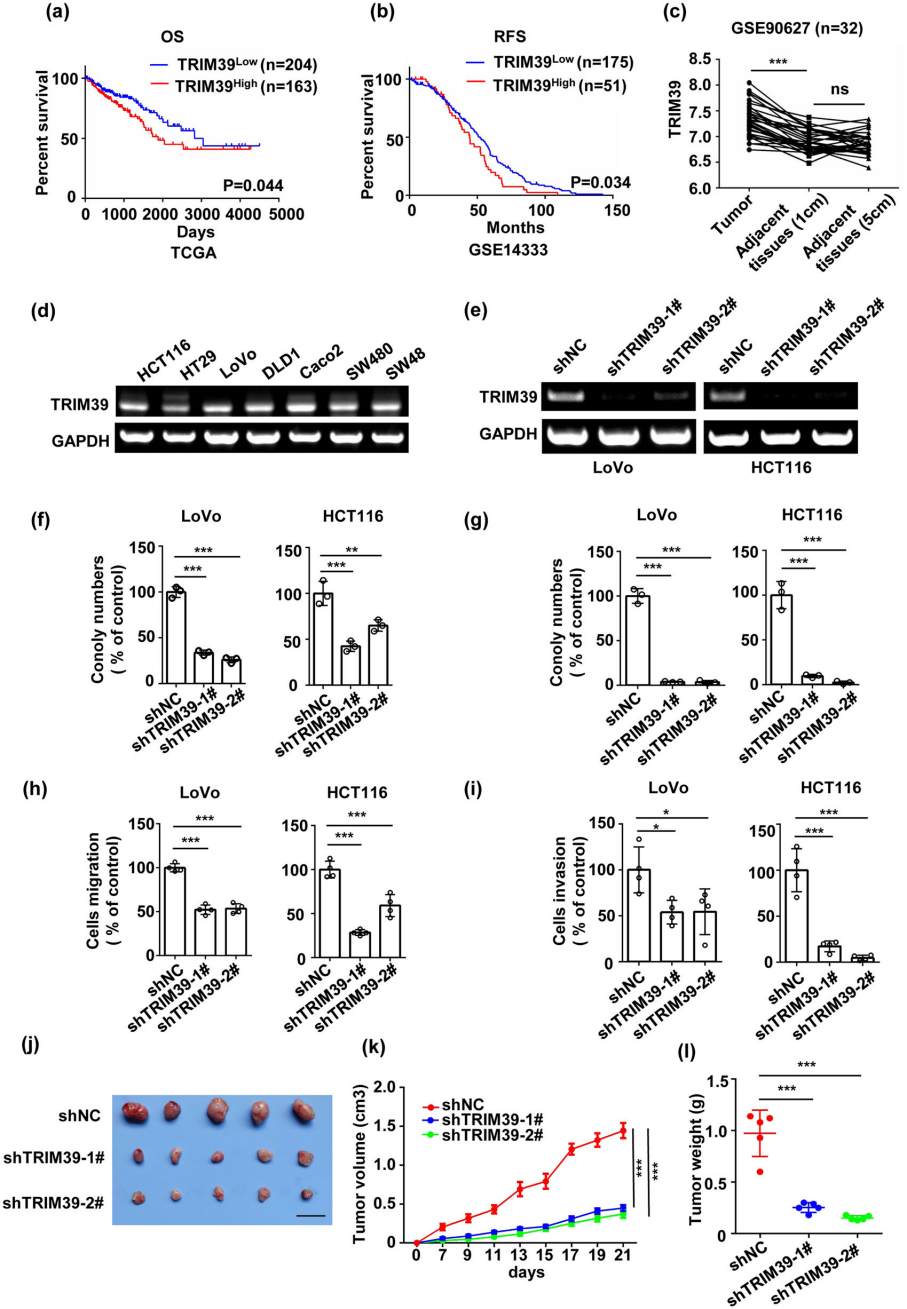
TRIM39 deficiency suppresses CRC progression in vitro and in vivo. **a** Kaplan–Meier survival analysis of overall survival (OS) based on TRIM39 expression in the CRC tissues from TCGA database. **b** Kaplan–Meier survival analysis of relapse-free survival (RFS) based on TRIM39 expression in the CRC tissues from GSE14333 database. **c** Analysis of TRIM39 mRNA levels in primary tumor and adjacent normal tissue in GSE90627 database. Two-way classification ANOVA. ***P < 0.001; ns no significance. **d** RT-PCR analysis of TRIM39 mRNA expression in HCT116, HT29, LoVo, DLD1, Caco2, SW480, and SW48 cells. **e** RT-PCR analysis of TRIM39 knockdown efficiency in LoVo and HCT116 cells infected with lentiviruses containing negative control shRNA (shNC) or two independent shRNAs against TRIM39. **f**, **g** The colony-formation assay (**f**) and soft agar colony-formation assay (**g**) of LoVo and HCT116 cells with TRIM39 stably knocked down. Data are shown as mean ± SD. *n* = 3 samples per group. One-way ANOVA. **P < 0.01; ***P < 0.001. **h**, **i** The migration assay (**h**) and invasion assay (**i**) of LoVo and HCT116 cells with TRIM39 stably knocked down. The average number of cells per field were calculated. Data are shown as mean ± SD. *n* = 3 samples per group, four fields per sample. One-way ANOVA. *P < 0.05; ***P < 0.001. **j**–**l** HCT116 cells (shNC, shTRIM39-1#, shTRIM39-2#) were subcutaneously injected into nude mice at a dose of 2 × 10^6^ cells per mouse. Representative images of the xenograft tumors (**j**), tumor growth curves (**k**), and tumor weight (**l**) are shown. *n* = 5 per group; data are shown as mean ± SD for tumor weight and mean ± SEM for tumor growth. Scale bar, 2 cm. One-way ANOVA. ***P < 0.001.

To assess the clinical significance of TRIM39 in CRC, we analyzed the clinical data from The Cancer Genome Atlas (TCGA; http://cancergenome.nih.gov) and GSE14333 databases^22^. Patients with low levels of TRIM39 had significantly improved overall survival (OS) (TCGA) and recurrence-free survival (GSE14333) (Fig. 1a, b). The mRNA levels of TRIM39 were higher in primary tumors of CRC patients compared to adjacent paired non-cancerous tissues in GSE90627 database^23^ (Fig. 1c). Higher TRIM39 expression was significantly correlated with deeper invasion, more lymph node metastasis, and advanced pathologic stages of CRC in TCGA (Table 1). Since TRIM39 antibodies generated by us or commercially purchased were not working with endogenous TRIM39, we conducted reverse transcription-polymerase chain reaction (RT-PCR) to detect TRIM39 expression and found that it was widely expressed in CRC cell lines (Fig. 1d). Functionally, TRIM39 knockdown by shRNA or siRNA could suppress cell colony formation, migration, and invasion abilities (Fig. 1e–i and Fig. S1a–i). Consistently, in vivo, TRIM39 knockdown inhibited tumor growth (Fig. 1j–l). Together, TRIM39 is critical to CRC progression in vitro and in vivo.

**Table 1:** Correlations between clinical pathological features and TRIM39 expression for CRC patients in TCGA.

Variable	*n*	TRIM39		*χ*^2^	*P*
		Low	High		
Age, years					
<65	170	96 (56.5%)	74 (43.5%)	0.1	0.753
≥65	197	108 (54.8%)	89 (45.2%)		
Gender					
Male	202	101 (50.0%)	101 (50.0%)	5.678	0.02
Female	165	103 (62.4%)	62 (37.6%)		
Depth of invasion					
T1/T2	67	49 (73.1%)	18 (26.9%)	10.366	0.002
T3/T4	299	154 (51.5%)	145 (48.5%)		
Lymph node metastases					
N0	201	127 (63.2%)	74 (36.8%)	10.004	0.002
N1/T2	163	76 (46.6%)	87 (53.4%)		
Distant metastasis					
M0	251	144 (57.4%)	107 (42.6%)	0.92	0.353
M1	50	25 (50.0%)	25 (50.0%)		
TNM stage					
I	56	42 (75.0%)	14 (25.0%)	17.557	0.001
II	132	82 (62.1%)	50 (37.9%)		
III	111	49 (44.1%)	62 (55.9%)		
IV	51	25 (49.0%)	26 (51.0%)		

### TRIM39 deficiency results in autophagosome accumulation in CRC

**Fig. 2:**
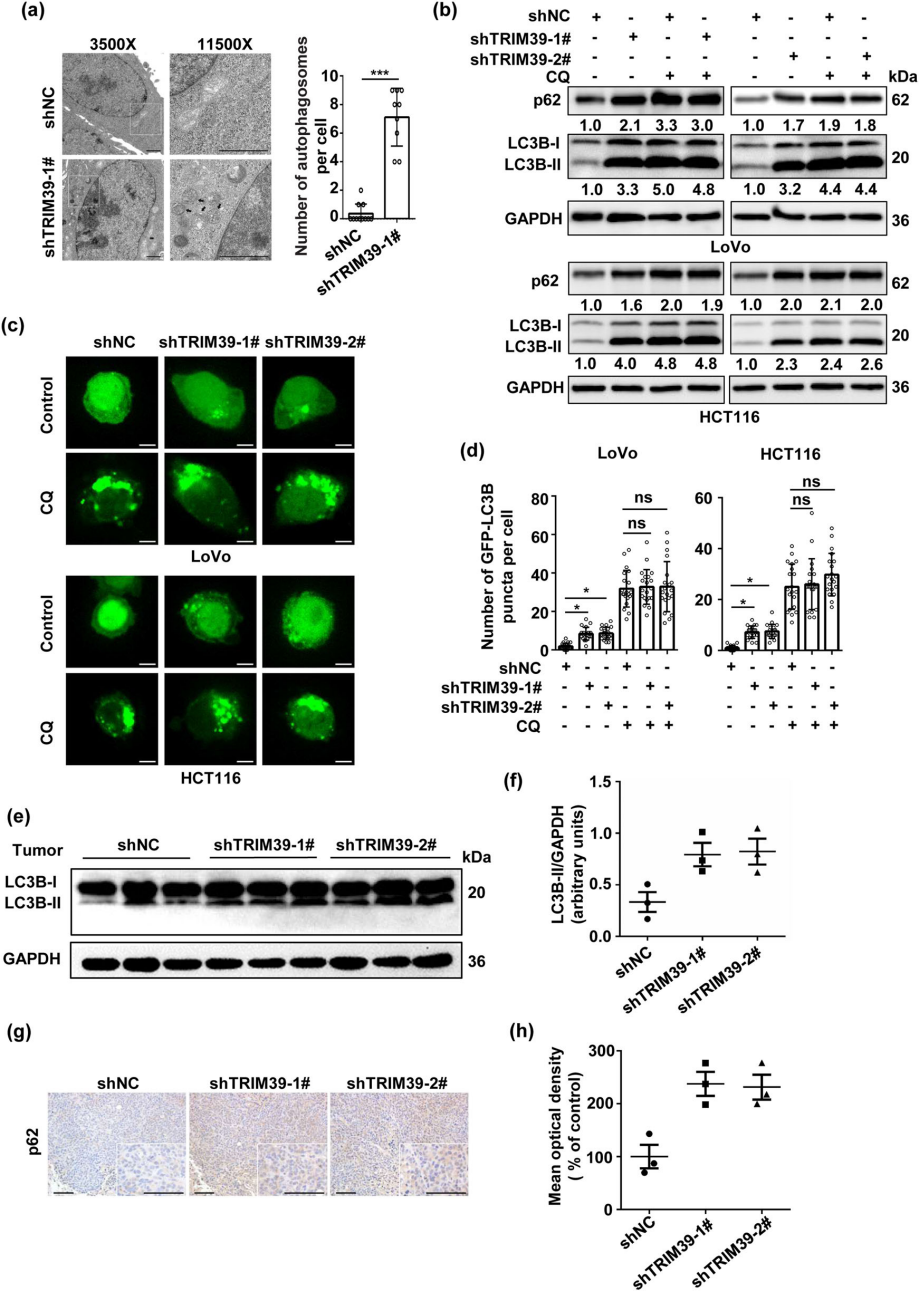
Knockdown of TRIM39 results in the accumulation of autophagosomes in CRC. **a** TEM analysis of TRIM39 stably knocked down HCT116 cells. Representative TEM images are shown. Black arrows indicate autophagic structures. The number of autophagic structures per cell was quantified. Data are shown as mean ± SD. Ten cells were scored. Scale bar, 1 μm. Student’s *t* test. ***P < 0.001. **b** Western blot analysis of p62 and LC3B-II levels in TRIM39 stably knocked down LoVo and HCT116 cells treated with CQ (50 nM) for 4 h. **c** Representative confocal microscopic images of GFP-LC3B puncta in TRIM39 stably knocked down LoVo and HCT116 cells. Scale bar, 5 μm. **d** Quantification of GFP-LC3B puncta per cell treated as in **c**. Data are shown as mean ± SD. Twenty cells were scored. One-way ANOVA. *P < 0.05; ns no significance. **e** Western blot analysis of LC3B-II levels of tumor tissues from different groups in Fig. 1j. **f** Quantification (arbitrary units) of LC3B-II levels relative to GAPDH in tumor tissues from **e**. Data are shown as mean ± SD. **g** Immunochemical staining for p62 of tumor tissues from different groups in Fig. 1j. Representative immunochemical staining images are shown. Scale bar, 50 μm. **h** Quantification of p62 levels (mean optical density) in tumor tissues from **g**.

We next explored the mechanisms underlying TRIM39’s oncological roles in CRC. TEM analysis showed that TRIM39 knockdown resulted in the appearance of autophagosome-like structures in CRC cells (Fig. 2a). In line with that, significantly increased number of GFP-LC3B puncta and elevated levels of endogenous LC3B-II were observed (Fig. 2b–d and Fig. S2d). This also occurred for endogenous LC3B-II in xenografted tumors (Fig. 1j and 2e, f). Such accumulated autophagosomes might be due to autophagy activation or inhibition of autophagosome degradation^24^. To differentiate these two possibilities, we used CQ, an inhibitor for autophagosome–lysosome fusion, to block autophagy process. CQ treatment abolished TRIM39 knockdown-induced autophagosome accumulation (Fig. 2b–d and Fig. S2d), while TRIM39 overexpression reduced autophagosome accumulation regardless of CQ treatment (Fig. S2a–c). SQSTM1/p62, an endogenous autophagy substrate degraded during autophagic process^24^, was accumulated upon TRIM39 knockdown (Fig. 2b, g, h). Together, these results suggest that autophagosome accumulation induced by TRIM39 knockdown is likely due to inhibition of autophagosome degradation.

### TRIM39 is required for autophagic flux in CRC

**Fig. 3:**
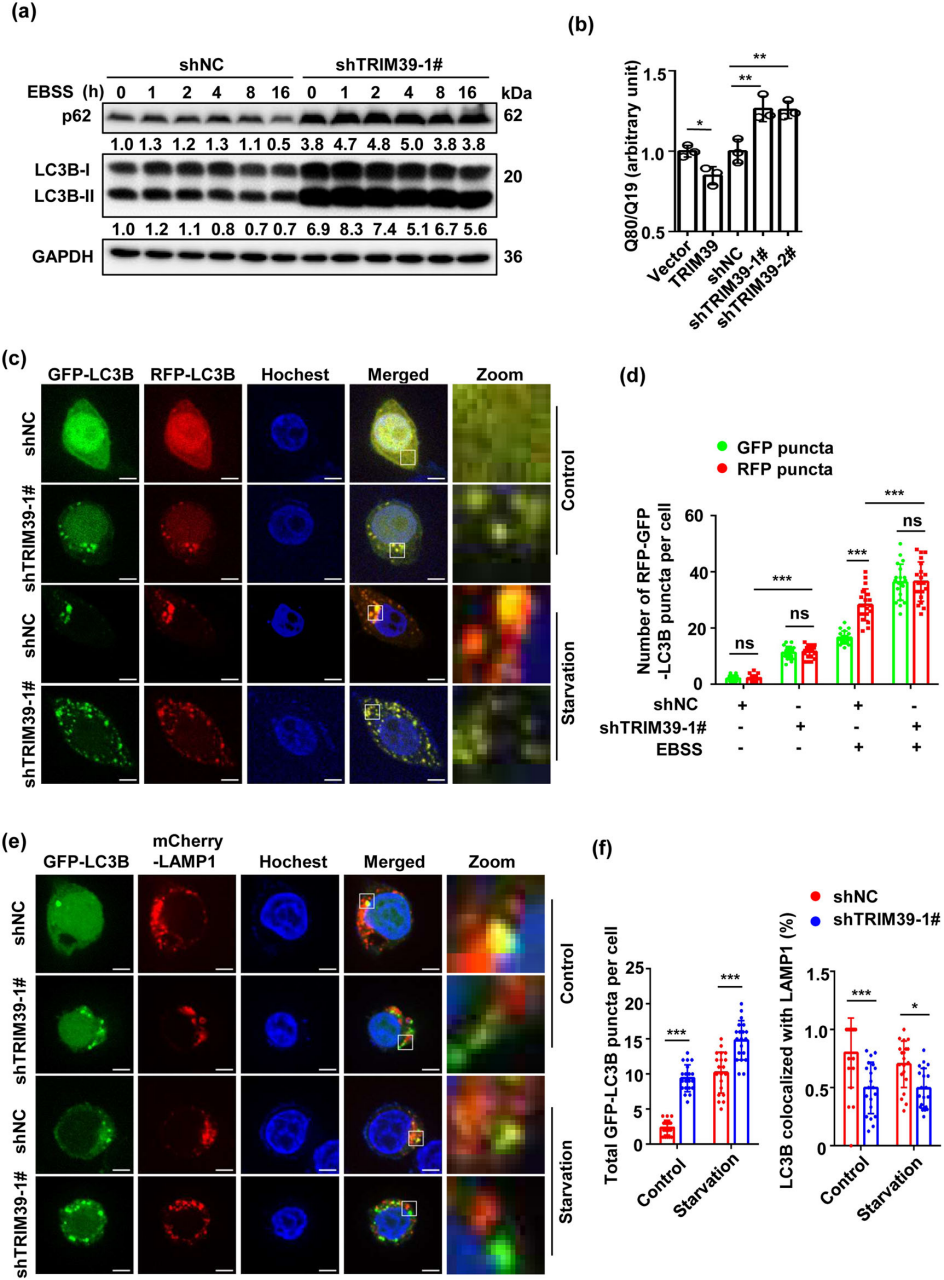
Knockdown of TRIM39 impairs autophagic flux in CRC cell lines. **a** TRIM39 stably knocked down LoVo cells and control cells (shNC) were treated with EBSS for the indicated times. The levels of p62 and LC3B-II were detected by western blot. **b** LoVo cells were co-transfected with the indicated plasmids and polyQ80-luciferase (or polyQ19-luciferase) for 48 h. TRIM39 stably knocked down LoVo cells were transfected with polyQ80-luciferase or polyQ19-luciferase for 48 h. Dual Luciferase Reporter System was used to analyze PolyQ80–luciferase/polyQ19-luciferase ratios. Degradation of polyQ19 was served as an internal control. The average value in empty vector-transfected cells or shNC cells was normalized as 1. Data are shown as mean ± SD. *n* = 3. Student’s *t* test for the first two columns and one-way ANOVA for the last three columns. *P < 0.05; **P < 0.01. **c** TRIM39 stably knocked down LoVo cells and control cells (shNC) were transfected with RFP-GFP-LC3B for 48 h and incubated in growth medium or EBSS for the last 2 h. RFP-GFP-LC3B distribution was observed by confocal microscopy. Representative confocal microscopy images are shown. Scale bar, 5 μm. **d** Quantification of GFP-positive and RFP-positive LC3-labeled puncta per cell treated as in **c**. Data are shown as mean ± SD. Twenty cells were scored. One-way ANOVA. ***P < 0.001; ns no significance. **e** TRIM39 stably knocked down LoVo cells and control cells (shNC) were co-transfected with GFP-LC3B and mCherry-LAMP1 for 48 h and incubated in growth medium or EBSS for the last 2 h. Co-localization of GFP-LC3B with mCherry-LAMP1 was observed by confocal microscopy. Representative confocal microscopy images are shown. Scale bar, 5 μm. **f** Quantification of the total GFP-LC3B puncta and the GFP-LC3B puncta colocalized with mCherry-LAMP1 per cell treated as in **e**. Data are shown as mean ± SD. Twenty cells were scored. One-way ANOVA. *P < 0.05; ***P < 0.001.

Next, we set to investigate the role of TRIM39 in autophagic flux. p62 was accumulated over time after TRIM39 knockdown (Fig. 3a). Consistently, polyQ80 aggregates (formed by polyglutamine with 80 repeats), often used as an exogenous substrate for studying autophagy^20,25^, were accumulated when TRIM39 was knocked down but effectively degraded in TRIM39-overexpressing cells (Fig. 3b). To further demonstrate TRIM39’s effect on autophagy, we utilized the cells transfected with RFP-GFP-LC3B plasmid that expressed green and red fluorescence proteins (GFP and RFP, respectively) simultaneously, which is a powerful tool to visually monitor autophagic flux^26^. When autophagosomes fuse with lysosomes, GFP fluorescence is quenched due to the acidic environment within lysosomes, whereas RFP signal persists. TRIM39 knockdown increased the number of yellow fluorescence puncta (Fig. 3c, d, non-starvation group), indicating autophagosome accumulation. During starvation-induced autophagy where both yellow and red-only puncta were increased (Fig. 3c, d, shNC in the starvation group), TRIM39 knockdown dramatically increased yellow puncta (Fig. 3c, d, shTRIM39 in the starvation group), suggesting an impaired autophagic flux. We then determined whether TRIM39 promoted autophagic flux by facilitating autophagosome–lysosome fusion using spotted GFP-LC3B as a marker for autophagosomes and LAMP1 for lysosomes. TRIM39 knockdown induced formation of more GFP-LC3B puncta that were, however, not colocalized with LAMP1 (Fig. 3e, f), suggesting a prevention of autophagosome–lysosome fusion. Another possible reason for the impaired late-stage autophagic process might be due to the decreased lysosome numbers or impaired lysosomal function. TRIM39 knockdown did not alter the puncta number and protein levels of lysosome marker LAMP1 (Fig. S3a–c). The LysoTracker Red, a specific pH probe, was employed to evaluate the acidic environment. Interestingly, TRIM39 deficiency promoted the acidification of lysosomes as evidenced by an increased fluorescence intensity of the pH probe (Fig. S3d, e), suggesting that late-stage autophagy inhibition is not linked to an impaired lysosomal acidification. We further detected the protein levels of mature cathepsin B (CTSB) and cathepsin D (CTSD), the most abundant lysosomal proteases critical for lysosomal degradation, and found that TRIM39 knockdown decreased the protein levels of mature CTSB and CTSD (Fig. S3f). Together, TRIM39 deficiency-inhibited autophagy flux may be attributable to the reduced autophagosome maturation and impaired lysosomal function.

### TRIM39 interacts with Rab7 and promotes its activity

**Fig. 4:**
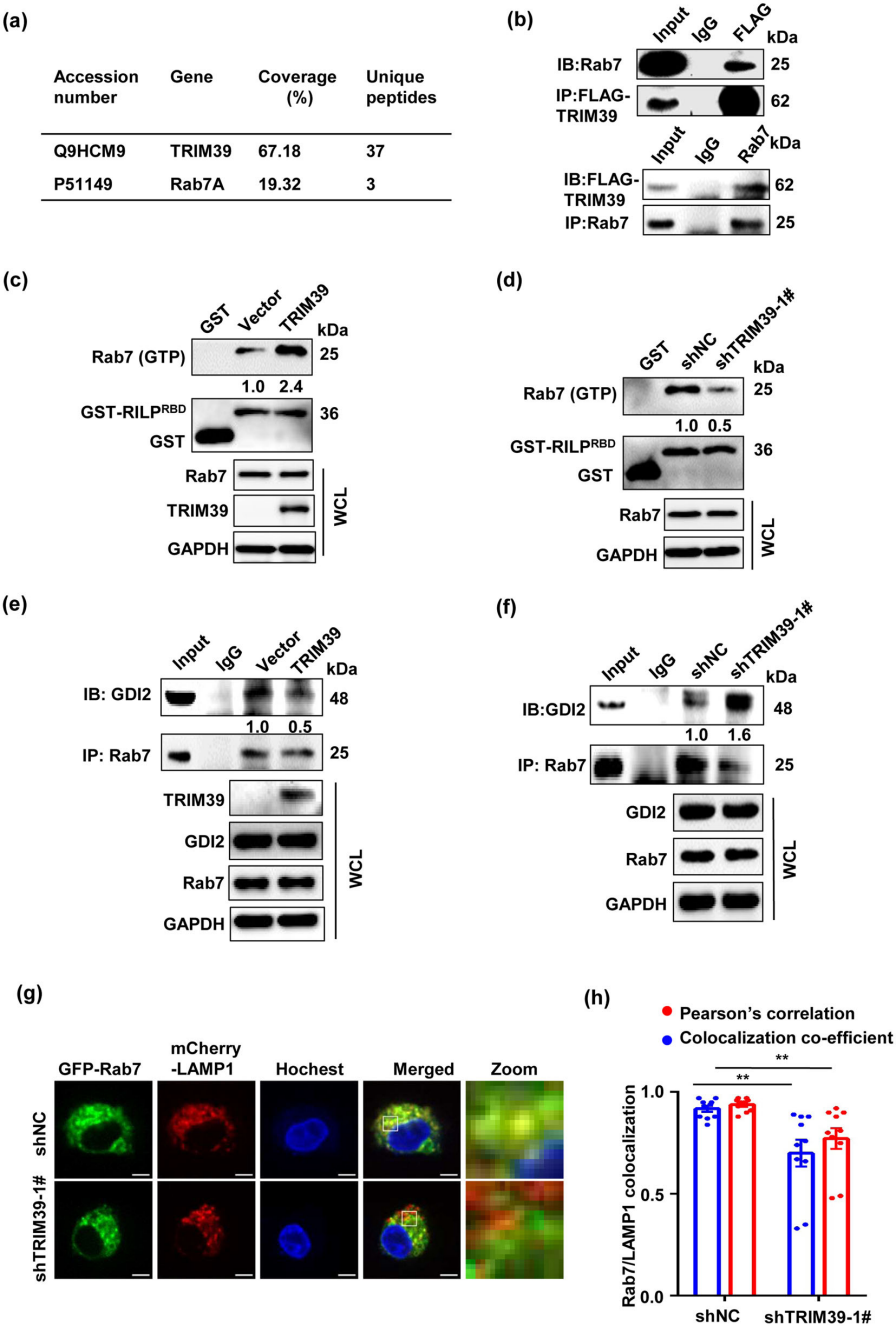
Rab7 is a binding partner of TRIM39. **a** HEK293T cells were transfected with empty vector or FLAG-TRIM39 for 48 h. Total cell lysates were subjected to immunoprecipitation with anti-FLAG antibody. Rab7 was identified via mass spectrometry. **b** HEK293T cells were transfected with FLAG-TRIM39 for 48 h. Total cell lysates were immunoprecipitated with anti-FLAG or anti-Rab7 antibodies. Rab7 and FLAG-TRIM39 were detected by western blot. **c**, **d** GST-RILP^RBD^ fusion protein immobilized on glutathione-Sepharose beads were incubated with Vector/FLAG-TRIM39-overexpressed LoVo cell lysates (**c**) or shNC/TRIM39 stably knocked down LoVo cell lysates (**d**) at 4 °C for 4 h. Rab7 and GST were detected in the washed beads by western blot. **e**, **f** Control (Vector) and FLAG-TRIM39-overexpressed LoVo cell lysates (**e**) or shNC and TRIM39 stably knocked down LoVo cell lysates (**f**) were extracted and immunoprecipitated using an anti-Rab7 antibody. Then the precipitates were examined with anti-GDI2. **g** shNC and TRIM39 stably knocked down LoVo cells were co-transfected with GFP-Rab7 and mCherry-LAMP1 for 48 h. Representative confocal microscopy images are shown. Scale bar, 5 μm. **h** Quantification of co-localization efficient and Pearson correlation of GFP-Rab7 and mCherry-LAMP1 treated as in **g**. 10 cells were analyzed. Data are shown as mean ± SEM. One-way ANOVA. **P < 0.01.

To gain mechanistic insight as to molecular mediators responsible for facilitating TRIM39-regulated autophagy, an immunoprecipitation-coupled mass spectrometry screen was performed. Rab7A (referred to as Rab7 in the following text), a small GTPase playing an important role in autophagosome maturation^27^, was identified as a TRIM39 interacting protein (Fig. 4a). The interaction was confirmed by reciprocal co-immunoprecipitation experiments (Fig. 4b). Since TRIM39 did not affect Rab7 protein levels, we determined whether TRIM39 regulated the GTPase activity of Rab7. Rab7-binding domain of Rab interacting lysosomal protein (RILP^RBD^) was often used as an indicator for Rab7 activity, as it interacts specifically with the GTP-bound, active form of Rab7^28,29^. TRIM39 overexpression and knockdown significantly elevated or attenuated the amount of active Rab7 precipitated by GST-RILP^RBD^, respectively (Fig. 4c, d). Consistently, TRIM39 overexpression and knockdown drastically reduced and increased the amount of precipitated GDI2 (Fig. 4e, f), a Rab7 chaperone only binding to inactive Rab7^30–32^, respectively, suggesting that TRIM39 promotes Rab7 activity. Moreover, TRIM39 knockdown reduced Rab7 localization in lyso-endosomes where it normally resided (Fig. 4g, h). Together, TRIM39 interacts with Rab7 and promotes its GTPase activity.

Since Rab7 is critical for the transit of early endosomes to late endosomes^33^, we hypothesized that TRIM39 was also involved in maturation of endosomes. Depletion of TRIM39 increased EEA1-positive vesicles (early endosome marker) and decreased CD63-positive vesicles (late endosome marker) (Fig. S4a–d). Knockdown of either TRIM39 or Rab7 increased the protein levels of EEA1 and reduced the levels of CD63 (Fig. S4e, f). Overexpression of Rab7 restored the alternation of EEA1 and CD63 in TRIM39-deficient cells (Fig. S4a–e). CTSB and CTSD are trafficked to the lysosomes through the early–late endosome–lysosome maturation process and become catalytically active (mature) within the acidic environment of lysosomes^34^. TRIM39 or Rab7 knockdown decreased the protein levels of mature CTSB and CTSD (Fig. S4e, f), suggesting that the trafficking of CTSB and CTSD from early endosomes to late ones is hampered. Overexpression of Rab7 could also restore decreased protein levels of mature CTSB and CTSD (Fig. S4e). These results suggest that TRIM39 promotes endosome maturation through Rab7.

### TRIM39 inhibits the ubiquitination of Rab7 at lysine 191 residue

**Fig. 5:**
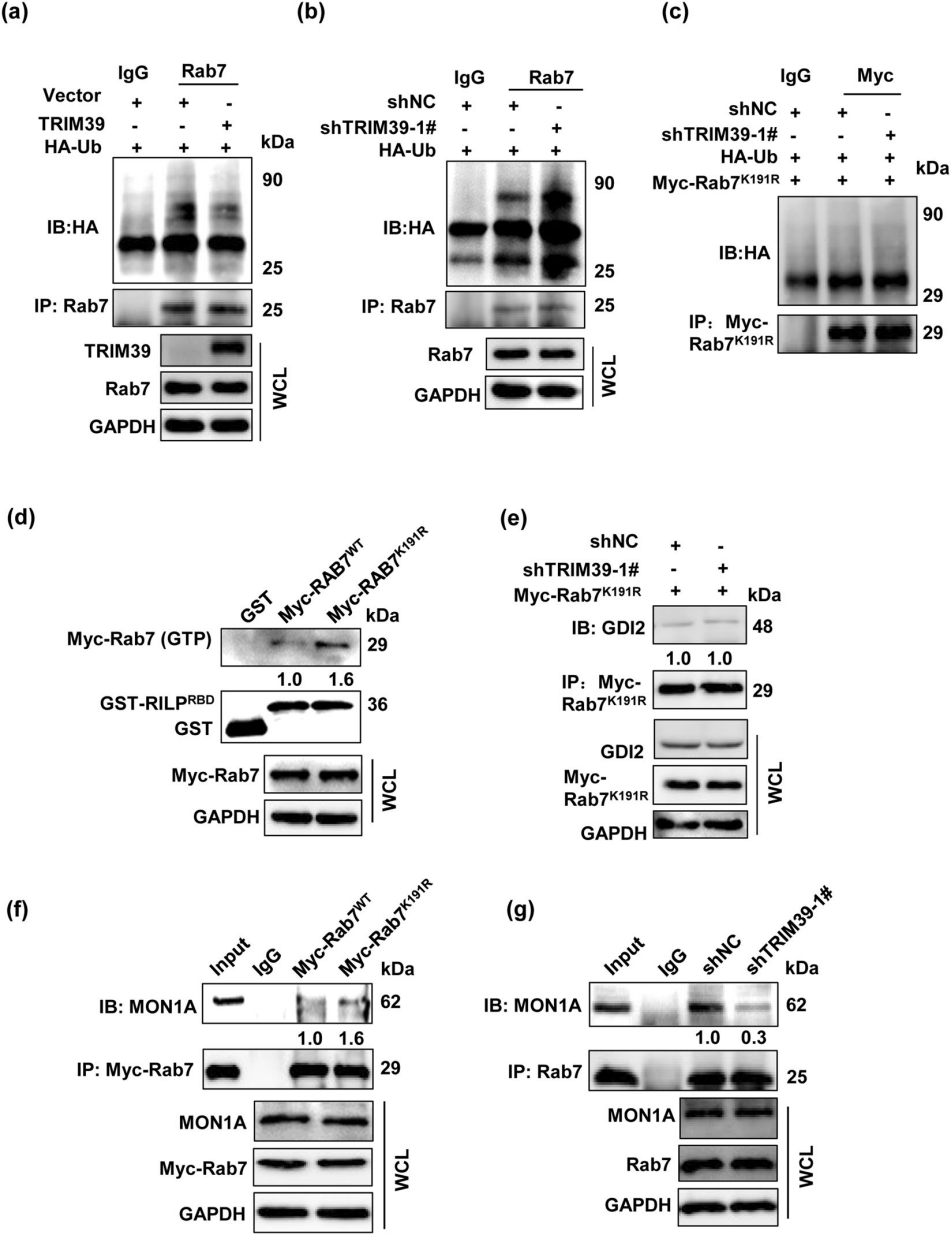
TRIM39 decreases the ubiquitination of Rab7 at lysine 191 residue. **a** Vector or FLAG-TRIM39 was co-transfected with HA-ubiquitin into HCT116 cells for 48 h. Cell lysates were extracted and immunoprecipitated using an anti-Rab7 antibody. Then the precipitates were examined with anti-HA. **b** shNC and TRIM39 stably knocked down HCT116 cells were transfected with HA-ubiquitin for 48 h. Cell lysates were extracted and immunoprecipitated using an anti-Rab7 antibody. Then the precipitates were examined with anti-HA. **c** Myc-Rab7^K191R^ was co-transfected with HA-ubiquitin into shNC or TRIM39 stably knocked down HCT116 cells for 48 h. Cell lysates were immunoprecipitated with anti-Myc antibody and immunoblotted with anti-HA and anti-Myc antibody. **d** GST-RILP^RBD^ fusion protein immobilized on glutathione-Sepharose beads were incubated with Myc-Rab7^WT^ or Myc-Rab7^K191R^ overexpressed HCT116 cell lysates at 4 °C for 4 h. Rab7 and GST were detected in the washed beads by western blot. **e** shNC and TRIM39 stably knocked down HCT116 cells were transfected with Myc-Rab7^K191R^ for 48 h. Cell lysates were immunoprecipitated with anti-Myc antibody and immunoblotted with anti-GDI2 and anti-Myc antibody. **f** HCT116 cells were transfected with Myc-Rab7^WT^ or Myc-Rab7^K191R^ for 48 h. Cell lysates were immunoprecipitated with anti-Myc antibody and immunoblotted with anti-MON1A and anti-Myc antibody. **g** shNC or TRIM39 stably knocked down HCT116 cell lysates were extracted and immunoprecipitated with anti-Rab7 antibody. MON1A was detected by western blot.

Rab7 activity can be influenced by ubiquitination^35,36^. We wondered whether TRIM39 ubiquitinated Rab7 to modulate its activity. Surprisingly, TRIM39 attenuated the ubiquitination of Rab7 instead of promoting its ubiquitination (Fig. 5a, b). Ubiquitinated proteome analysis showed that K38, K191, and K126 were the possible ubiquitinated lysine residues of Rab7^37,38^. We constructed Myc-tagged Rab7 K-to-R mutated plasmids (K38R, K191R, and K126R) to identify specific ubiquitination sites modulated by TRIM39. Rab7^K38R^ and Rab7^K126R^ but not Rab7^K191R^ increased Rab7 ubiquitination when TRIM39 was knocked down (Fig. 5c and Fig. S5a, b), and the activity of Rab7^K191R^ mutant was elevated and no longer affected by TRIM39 (Fig. 5d, e), indicating that K191 is the ubiquitination site influenced by TRIM39. We further found that Rab7^K191R^ mutant did not affect its interaction with TRIM39 but was co-isolated more with MON1A, a component of GEF toward Rab7 (Fig. 5f and Fig. S5c). TRIM39 knockdown also resulted in the loss of Rab7 binding to MON1A (Fig. 5g). Thus, TRIM39 promotes Rab7 activity via inhibiting its ubiquitination at lysine 191 residue and facilitating its interaction with MON1A. Moreover, such TRIM39-mediated promotion was independent of TRIM39’s E3 ubiquitin ligase activity, as evidenced by the fact that RING domain-truncated TRIM39 could still promote Rab7 activity and reduced autophagosome accumulation (Fig. S5d, e).

### TRIM39 promotes CRC progression through Rab7

**Fig. 6:**
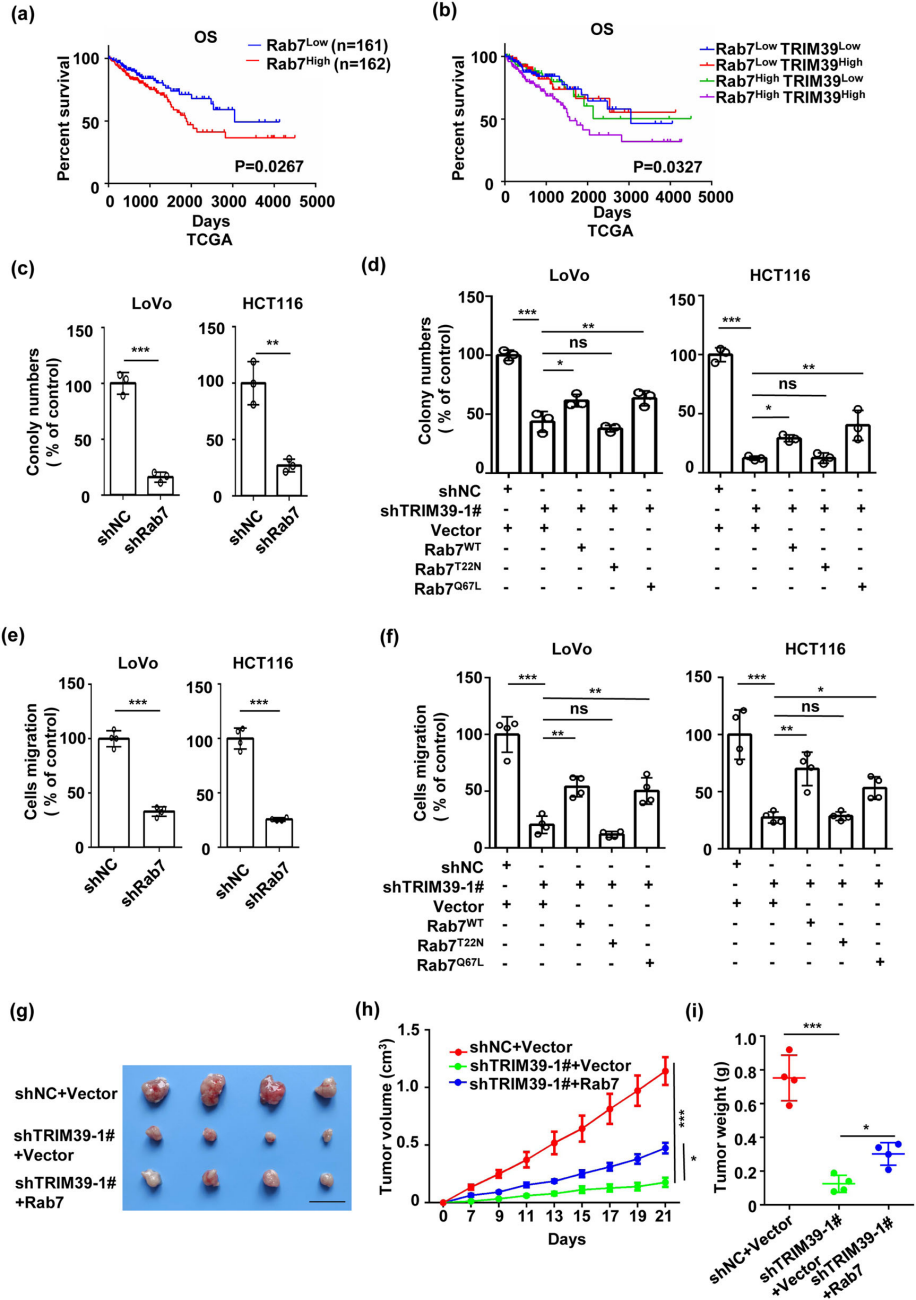
TRIM39 depletion suppresses CRC through inhibiting Rab7 activity. **a** Kaplan–Meier survival analysis of overall survival (OS) based on Rab7 expression in the CRC tissues from TCGA database. **b** Kaplan–Meier survival analysis of overall survival (OS) based on TRIM39 and Rab7 expression in the CRC tissues from TCGA database. Patients were classified as TRIM39^High^Rab7^High^, TRIM39^Low^Rab7^High^, TRIM39^High^Rab7^Low^, and TRIM39^Low^Rab7^Low^ groups. **c** The colony-formation assay of LoVo and HCT116 cells with Rab7 stably knocked down. Data are shown as mean ± SD. *n* = 3 samples per group. Student’s *t* test. **P < 0.01; ***P < 0.001. **d** Colony-formation assay of TRIM39 stably knocked down LoVo and HCT116 cells infected with Empty vector, Rab7^WT^, Rab7^T22N^, and Rab7^Q67L^ overexpression lentivirus. Data are shown as mean ± SD. *n* = 3 samples per group. One-way ANOVA. *P < 0.05; **P < 0.01; ***P < 0.001; ns no significance. **e** The migration assay of LoVo and HCT116 cells with Rab7 stably knocked down. The average number of cells per field was calculated. Data are shown as mean ± SD. *n* = 3 samples per group, four fields per sample. Student’s *t* test. ***P < 0.001. **f** The migration assay of TRIM39 stably knocked down LoVo and HCT116 cells infected with Empty vector, Rab7^WT^, Rab7^T22N^, and Rab7^Q67L^ overexpression lentivirus. The average number of cells per field was calculated. Data are shown as mean ± SD. *n* = 3 samples per group, four fields per sample. One-way ANOVA. *P < 0.05; **P < 0.01; ***P < 0.001; ns no significance. **g**–**i** HCT116 cells (shNC, shTRIM39-1#) were infected with Vector or Rab7 overexpression lentivirus and then subcutaneously injected into nude mice. Representative images of the xenograft tumors (**g**), tumor growth curves (**h**), and tumor weight (**i**) are shown. *n* = 4 per group; Data are shown as mean ± SD for tumor weight and mean ± SEM for tumor growth. Scale bar, 2 cm. One-way ANOVA. *P < 0.05; ***P < 0.001.

Rab7 acts as a pro-tumorigenic or a tumor-inhibiting gene depending on cancer types^39–41^. However, the specific roles of Rab7 in CRC remained unclear. While CRC patients with high levels of Rab7 had poorer OS (Fig. 6a), patients with high levels of both TRIM39 and Rab7 (TRIM39^High^Rab7^High^) had the poorest OS (Fig. 6b), suggesting that Rab7 and TRIM39 may be functionally synergized during CRC progression. Rab7 knockdown inhibited tumor growth and CRC cell migration in vitro and in vivo, similar to the phenotypes observed for TRIM39 knockdown (Fig. 6c, e and Fig. S6b–j). Wild-type Rab7 and consecutively activated mutant Rab7 (Rab7^Q67L^, GTP-bound form) but not consecutively inactivated mutant Rab7 (Rab7^T22N^, GDP-bound form) could rescue the growth and migration inhibition induced by TRIM39 knockdown (Fig. 6d, f and Fig. S7a, b). Rab7 overexpression reversed in vivo tumor suppression caused by TRIM39 knockdown (Fig. 6g–i). Thus, TRIM39 promotes CRC progression in a Rab7 activity-dependent manner.

### TRIM39 promotes autophagic flux in a Rab7 activity-dependent fashion

**Fig. 7:**
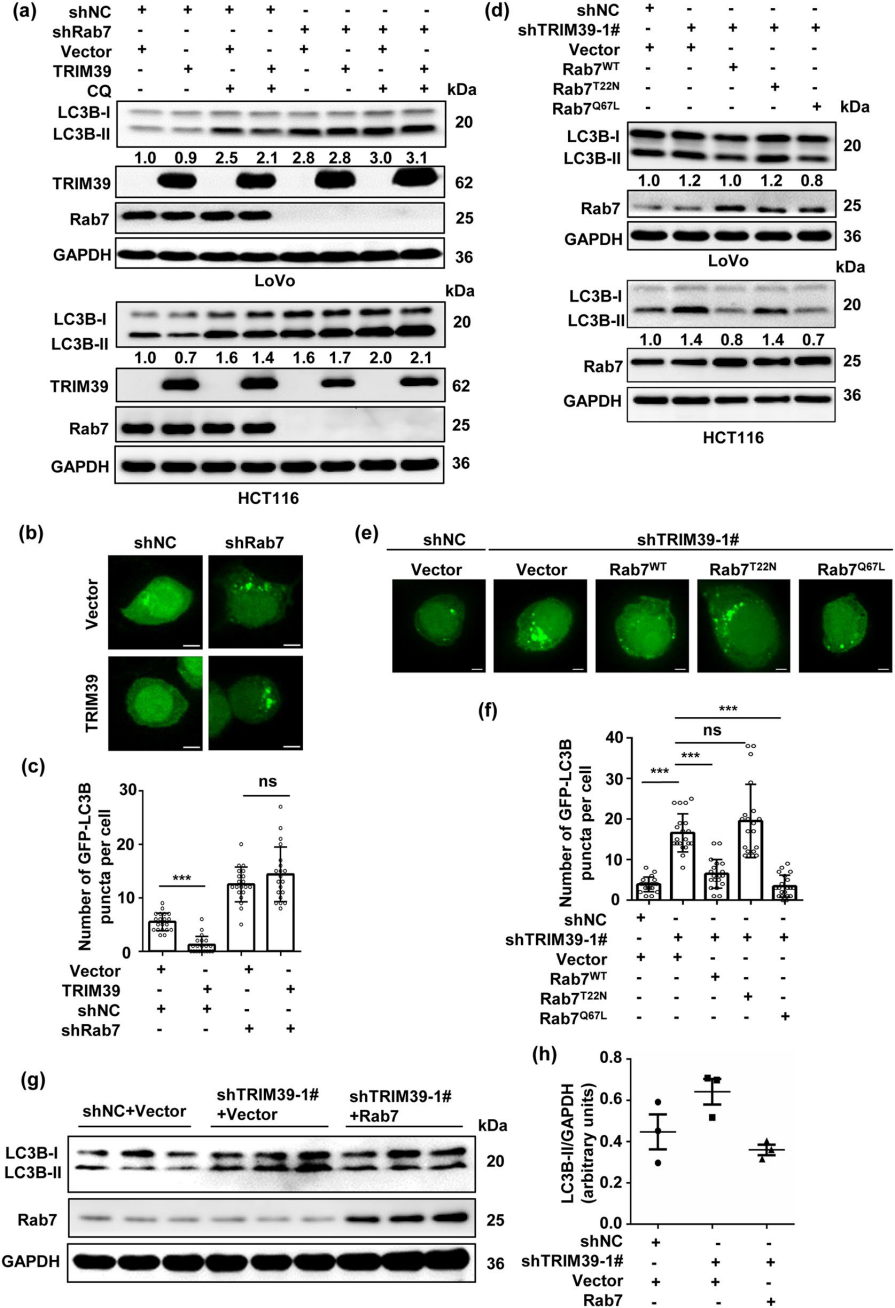
TRIM39 promotes autophagic flux via Rab7. **a** Rab7 stably knocked down and control LoVo and HCT116 cells were transfected with empty vector or FLAG-TRIM39 plasmids for 48 h and then treated with or without CQ (50 nM) for the last 4 h. The levels of LC3B-II were detected by western blot. **b** Representative confocal microscopic images of GFP-LC3B puncta in Rab7 stably knocked down LoVo cells transfected with empty vector or FLAG-TRIM39 for 48 h. Scale bar, 5 μm. **c** Quantification of GFP-LC3B puncta per cell treated as in **b**. Data are shown as mean ± SD. Twenty cells were scored. One-way ANOVA. ***P < 0.001; ns no significance. **d** TRIM39 stably knocked down LoVo and HCT116 cells were infected with empty vector, Rab7^WT^, Rab7^T22N^, and Rab7^Q67L^ overexpression lentivirus. The levels of LC3B-II were detected by western blot. **e** TRIM39 stably knocked down LoVo cells were infected with empty vector, Rab7^WT^, Rab7^T22N^, and Rab7^Q67L^ overexpression lentivirus. GFP-LC3B puncta was observed by confocal microscopy. Representative images are shown. Scale bar, 5 μm. **f** Quantification of GFP-LC3B puncta per cell treated as in **e**. Data are shown as mean ± SD. Twenty cells were scored. One-way ANOVA. ***P < 0.001; ns no significance. **g** Western blot analysis of LC3B-II levels of tumor tissues from different groups in Fig. 6g. **h** Quantification (arbitrary units) of LC3B-II levels relative to GAPDH in tumor tissues from **g**. Data are shown as mean ± SD.

Rab7 plays a crucial role in autophagosome maturation^42–44^. Consistently, Rab7 knockdown led to the accumulation of both LC3B-II and p62 in human CRC cells (Fig. S6a) and abolished TRIM39 overexpression-enhanced autophagic degradation of LC3B-II (Fig. 7a–c), suggesting that TRIM39’s regulation on autophagy is dependent on Rab7 activity. Supporting this notion, wild-type Rab7 and consecutively active Rab7 (Rab7^Q67L^) were able to decrease the accumulation of GFP-LC3B puncta and the elevated endogenous LC3B-lI levels induced by TRIM39 knockdown (Fig. 7d–f). This was verified in vivo (Fig. 7g, h). Taken together, TRIM39 promotes autophagic flux in a Rab7 activity-dependent fashion.

### TRIM39 deficiency suppresses CRC progression through inhibiting autophagic degradation of p53

**Fig. 8:**
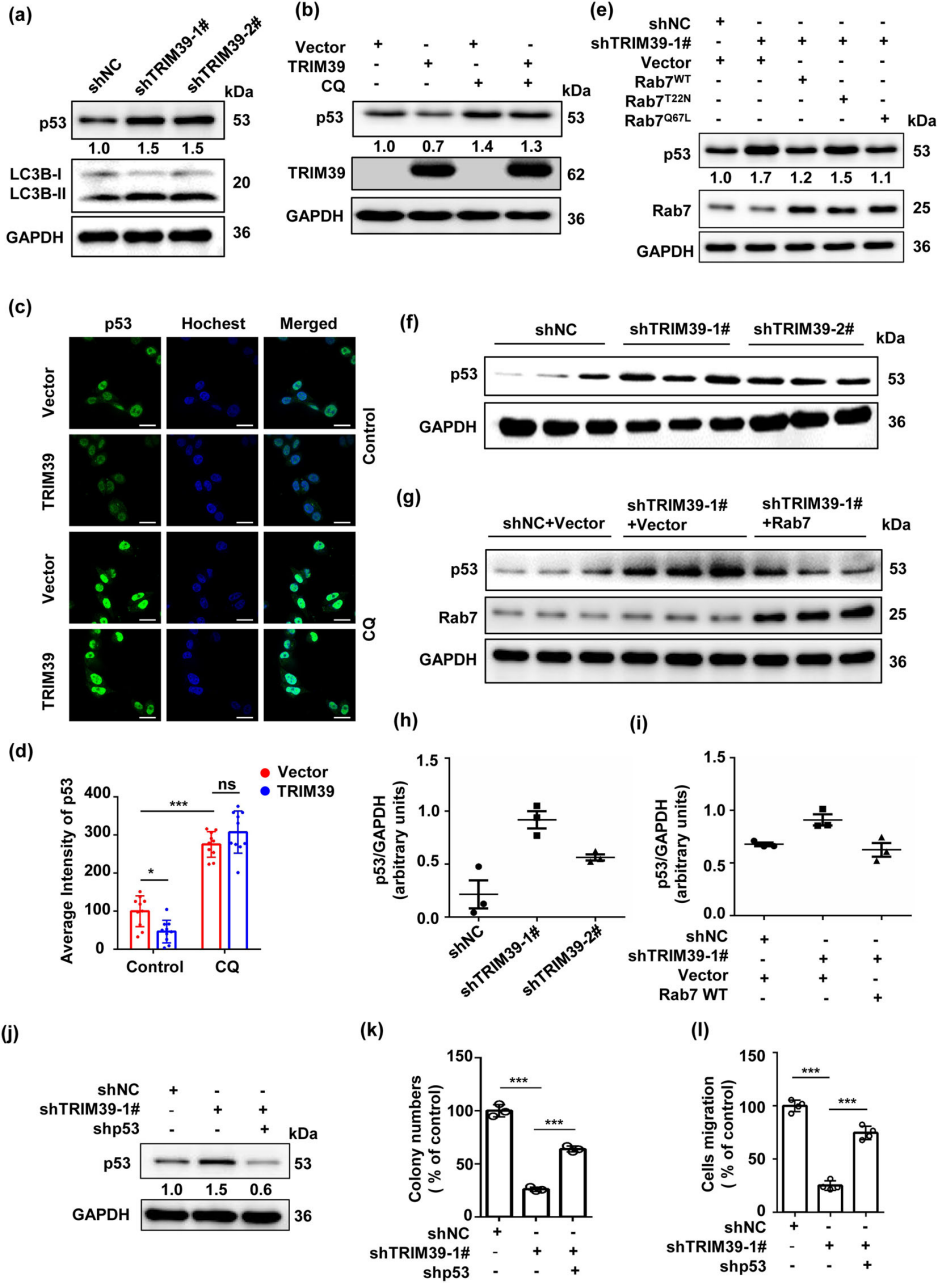
Inhibiting autophagic degradation of p53 reverses the TRIM39 deficiency-suppressed CRC progression. **a** Western blot assay of p53 levels in TRIM39 stably knocked down HCT116 cells. **b** HCT116 cells were transfected with empty vector or FLAG-TRIM39 for 48 h and treated with or without CQ (50 nM) for the last 4 h. The levels of p53 were detected by western blot. **c** HCT116 cells were transfected with empty vector or FLAG-TRIM39 for 48 h and treated with or without CQ (50 nM) for the last 4 h. Cells were fixed and immunofluorescently stained for p53. Representative confocal microscopic images are shown. Scale bar, 20 μm. **d** The fluorescence intensity of p53 in **c** was calculated. Data are shown as mean ± SD. Ten images were scored. One-way ANOVA. *P < 0.05; ***P < 0.001; ns no significance. **e** TRIM39 stably knocked down HCT116 cells were infected with empty vector, Rab7^WT^, Rab7^T22N^, and Rab7^Q67L^ overexpression lentivirus. The levels of p53 were detected by western blot. **f** Western blot analysis of p53 levels of tumor tissues from different groups in Fig. 1j. **g** Western blot analysis of p53 levels of tumor tissues from different groups in Fig. 6g. **h** Quantification (arbitrary units) of p53 levels relative to GAPDH in tumor tissues from **f**. Data are shown as mean ± SD. **i** Quantification (arb**i**trary units) of p53 levels relative to GAPDH in tumor tissues from **g**. Data are shown as mean ± SD. **j** Western blot assay of p53 levels in TRIM39-depleted HCT116 cells with p53 stably knocked down. **k** The colony-formation assay of TRIM39-depleted HCT116 cells with p53 stably knocked down. Data are shown as mean ± SD. *n* = 3 samples per group. One-way ANOVA. ***P < 0.001. **l** The migration assay of TRIM39-depleted HCT116 cells with p53 stably knocked down. The average number of cells per field was calculated. Data are shown as mean ± SD. *n* = 3 samples per group, four fields per sample. One-way ANOVA. ***P < 0.001.

Consistent with previous study showing that TRIM39 could directly bind to and ubiquitinate p53, leading to p53 proteasomal degradation^19^, TRIM39 indeed negatively regulated the protein levels of p53 in human CRC cells (Fig. 8a, b and Fig. S8a). Intriguingly, such TRM39’s negative regulation on p53 could be partly reversed by the autophagy inhibitor CQ (Fig. 8b–d), indicating an involvement of autophagic degradation. Similar regulations on p53 could be observed for Rab7 overexpression or knockdown (Fig. S8b, c). Moreover, Rab7 overexpression abolished p53 accumulation induced by TRIM39 knockdown in vitro and in vivo (Fig. 8e–i), suggesting TRIM39’s dependence on Rab7 in autophagic degradation of p53. Further, p53 knockdown could restore CRC cells’ abilities in colony formation and migration impaired by TRIM39 knockdown (Fig. 8j–l and Fig. S8d, e). Collectively, these findings indicate that TRIM39 deficiency suppresses CRC progression by inhibiting autophagic degradation of p53.

## Discussion

Several members of the TRIM protein family are important regulators of CRC^45–48^. TRIM59 facilitates proliferation of CRC and promotes metastasis via the phosphoinositide 3-kinase/AKT pathway^49^. Downregulation of TRIM58 is associated with poor outcomes in CRC and enhances CRC cell invasion^50^. However, the functions of TRIM39 and the underlying mechanisms remain unexplored in CRC. In this study, we showed that TRIM39 was upregulated in CRC and significantly associated with poor survival of CRC patients. TRIM39 deficiency was able to inhibit CRC development in vitro and in vivo, suggesting that TRIM39 acts as an oncogene in CRC.

TRIM proteins are considered to function as receptors for cargo recognition or as platforms for core regulators of autophagosome formation^12,51^. TRIM5α promotes autophagy initiation by acting as a platform to assemble activated ULK1 and Beclin 1^51^. TRIM16 facilitates protein aggregate assembly and degradation by positively regulating the p62–NRF2 axis^52^. However, no TRIM members were reported to regulate the late stage of autophagic flux (autophagosome maturation). Here we showed that knockdown of TRIM39 resulted in the accumulation of autophagosomes, which was due to the impairment of autophagosome maturation and lysosomal function. The decreased levels of mature CTSB/D might be due to the reason that they acted as cargoes of endosomes and their transportation to lysosomes was inhibited upon TRIM39 knockdown. We identified that Rab7, a small GTPase cycling between GTP-bound functional form and GDP-bound non-functional form^33^, was the binding partner of TRIM39. Rab7 plays an important role in the final maturation of autophagosomes to autolysosomes and its activity is necessary for this process^42–44,53^. As an E3-ubiquitin ligase, TRIM39 did not change the protein levels of Rab7 but promoted the GTPase activity of Rab7. When Rab7 is recruited to endosomal membrane, GDI (GDP dissociation inhibitor) displacement factor (GDF) displaces GDI and presents it to GEF (GDP/GTP exchange factor) for activation^54^. TRIM39 promotes Rab7 binding to MONA1, a component of GEF, to upregulate Rab7’s activity. TRIM39 promoted autophagic flux in a Rab7 activity-dependent manner.

Ubiquitination as an important posttranslational protein modification has multiple roles, such as targeting proteins for proteasomal-mediated degradation^55^. TRIM39 directly bound to ubiquitinate p53 and promote its degradation^19^. Meanwhile, Rab7 activity can also be regulated by ubiquitination^35^. While parkin increased the protein levels and activity of Rab7 by promoting its ubiquitination on lysine 38 residue^56^, USP32 deubiquitinated Rab7 on lysine 191 residue to increase its activity without affecting its protein levels^36^. Similarly, TRIM39 promoted Rab7 activity also by inhibiting Rab7 ubiquitination on lysine 191 residue. Given that no interactions between Rab7 and USP32 in CRC were found (data not shown), other molecules might be involved in TRIM39’s regulation on Rab7 ubiquitination, which awaits further exploration.

Compared with defined roles in autophagy, the function of Rab7 in tumors remains controversial, as both pro-tumorigenic and onco-suppressor functions were reported^39–41^. Rab7 acted as an oncogene in several cancer types, such as cervix squamous carcinoma and lung cancer^57^, but as a tumor inhibitor in glioblastoma and prostate cancers^41,58^, suggesting that the functions of Rab7 are dependent on types of cancer. As for CRC, our results showed that high expression of Rab7 was correlated with poor prognosis in CRC patients and Rab7 knockdown suppressed CRC progression, which were similar to what were observed for TRIM39. Our molecular, biochemical, animal-level, and clinical data provide compelling evidence demonstrating that this is because the function of TRIM39 in CRC was largely dependent on Rab7 activity. Thus, we identify a TRIM39–Rab7 axis participating in regulating both autophagy and CRC progression, which may be a novel target for CRC treatment.

Previous studies revealed that TRIM39 had a relationship with the tumor-suppressor gene p53, but the results in these articles were contradictory. A study showed that TRIM39 did not affect the protein levels of p53 but decreased the protein levels of p21, which is a downstream target of p53^17^. In contrast, another study demonstrated that TRIM39 interacted with p53 and promoted its proteasome-mediated degradation^19^. Similar to this study, we also found that TRIM39 downregulated p53 protein levels in CRC but through autophagy. Moreover, autophagically degraded p53 accounted for TRIM39’s promoting effect on CRC.

In conclusion, we demonstrate for the first time that TRIM39 regulates autophagy and tumor progression through modulating Rab7 activity. TRIM39 interacts and inhibits the ubiquitination of Rab7 on K191 residue to promote its activity. TRIM39 deficiency suppresses CRC progression via inhibiting autophagic degradation of p53. Our findings underscore the role of TRIM39 in CRC, contribute to the understanding of Rab7 activity regulation, and highlight the importance of autophagy in CRC progression. The work may provide new therapeutic targets for CRC therapy.

## Supplementary information

suppl. info.

## References

[1] Bray F (2018). Global cancer statistics 2018: GLOBOCAN estimates of incidence and mortality worldwide for 36 cancers in 185 countries. CA Cancer J. Clin..

[2] Koveitypour Z (2019). Signaling pathways involved in colorectal cancer progression. Cell Biosci..

[3] Zoratto F (2014). Focus on genetic and epigenetic events of colorectal cancer pathogenesis: implications for molecular diagnosis. Tumour Biol..

[4] Devenport, S. N. & Shah, Y. M. Functions and implications of autophagy in colon cancer. *Cells* **8**, 1349 (2019). 10.3390/cells8111349 PMC6912527 31671556

[5] He C, Klionsky DJ (2009). Regulation mechanisms and signaling pathways of autophagy. Annu. Rev. Genet..

[6] McAfee Q (2012). Autophagy inhibitor Lys05 has single-agent antitumor activity and reproduces the phenotype of a genetic autophagy deficiency. Proc. Natl Acad. Sci. USA.

[7] Wiersma VR (2015). The epithelial polarity regulator LGALS9/galectin-9 induces fatal frustrated autophagy in KRAS mutant colon carcinoma that depends on elevated basal autophagic flux. Autophagy.

[8] Zheng Y (2009). Chloroquine inhibits colon cancer cell growth in vitro and tumor growth in vivo via induction of apoptosis. Cancer Investig..

[9] Liu J (2019). Blocking AMPK/ULK1-dependent autophagy promoted apoptosis and suppressed colon cancer growth. Cancer Cell Int..

[10] Sasaki K (2010). Chloroquine potentiates the anti-cancer effect of 5-fluorouracil on colon cancer cells. BMC Cancer.

[11] Zhang S (2019). SNX10 (sorting nexin 10) inhibits colorectal cancer initiation and progression by controlling autophagic degradation of SRC. Autophagy.

[12] Hatakeyama S (2017). TRIM family proteins: roles in autophagy, immunity, and carcinogenesis. Trends Biochem. Sci..

[13] Watanabe M, Hatakeyama S (2017). TRIM proteins and diseases. J. Biochem..

[14] Tan P (2018). TRIM59 promotes breast cancer motility by suppressing p62-selective autophagic degradation of PDCD10. PLoS Biol..

[15] Pan X, Chen Y, Shen Y, Tantai J (2019). Knockdown of TRIM65 inhibits autophagy and cisplatin resistance in A549/DDP cells by regulating miR-138-5p/ATG7. Cell Death Dis..

[16] Suzuki M (2016). TRIM39 negatively regulates the NFkappaB-mediated signaling pathway through stabilization of Cactin. Cell Mol. Life Sci..

[17] Zhang L (2012). TRIM39 regulates cell cycle progression and DNA damage responses via stabilizing p21. Proc. Natl Acad. Sci. USA.

[18] Lee SS (2009). TRIM39 is a MOAP-1-binding protein that stabilizes MOAP-1 through inhibition of its poly-ubiquitination process. Exp. Cell Res..

[19] Zhang L, Huang NJ, Chen C, Tang W, Kornbluth S (2012). Ubiquitylation of p53 by the APC/C inhibitor Trim39. Proc. Natl Acad. Sci. USA.

[20] Ju JS (2009). Quantitation of selective autophagic protein aggregate degradation in vitro and in vivo using luciferase reporters. Autophagy.

[21] Liang C (2008). Beclin1-binding UVRAG targets the class C Vps complex to coordinate autophagosome maturation and endocytic trafficking. Nat. Cell Biol..

[22] Jorissen RN (2009). Metastasis-associated gene expression changes predict poor outcomes in patients with Dukes stage B and C colorectal cancer. Clin. Cancer Res..

[23] Guo H (2017). Integrated transcriptomic analysis of distance-related field cancerization in rectal cancer patients. Oncotarget.

[24] Klionsky DJ (2012). Guidelines for the use and interpretation of assays for monitoring autophagy. Autophagy.

[25] Ravikumar B, Duden R, Rubinsztein DC (2002). Aggregate-prone proteins with polyglutamine and polyalanine expansions are degraded by autophagy. Hum. Mol. Genet..

[26] Kimura S, Noda T, Yoshimori T (2007). Dissection of the autophagosome maturation process by a novel reporter protein, tandem fluorescent-tagged LC3. Autophagy.

[27] Zhao YG, Zhang H (2019). Autophagosome maturation: an epic journey from the ER to lysosomes. J. Cell Biol..

[28] Cantalupo G, Alifano P, Roberti V, Bruni CB, Bucci C (2001). Rab-interacting lysosomal protein (RILP): the Rab7 effector required for transport to lysosomes. EMBO J..

[29] Wu M, Wang T, Loh E, Hong W, Song H (2005). Structural basis for recruitment of RILP by small GTPase Rab7. EMBO J..

[30] Rak A (2003). Structure of Rab GDP-dissociation inhibitor in complex with prenylated YPT1 GTPase. Science.

[31] Sivars U, Aivazian D, Pfeffer SR (2003). Yip3 catalyses the dissociation of endosomal Rab-GDI complexes. Nature.

[32] Seabra MC, Wasmeier C (2004). Controlling the location and activation of Rab GTPases. Curr. Opin. Cell Biol..

[33] Guerra, F. & Bucci, C. Multiple roles of the small GTPase Rab7. *Cells* **5**, 34 (2016). 10.3390/cells5030034 PMC5040976 27548222

[34] Brix K, Dunkhorst A, Mayer K, Jordans S (2008). Cysteine cathepsins: cellular roadmap to different functions. Biochimie.

[35] Modica G, Lefrancois S (2020). Post-translational modifications: How to modulate Rab7 functions. Small GTPases.

[36] Sapmaz A (2019). USP32 regulates late endosomal transport and recycling through deubiquitylation of Rab7. Nat. Commun..

[37] Wagner SA (2011). A proteome-wide, quantitative survey of in vivo ubiquitylation sites reveals widespread regulatory roles. Mol. Cell. Proteomics.

[38] Kim W (2011). Systematic and quantitative assessment of the ubiquitin-modified proteome. Mol. Cell.

[39] Wang T (2012). A role of Rab7 in stabilizing EGFR-Her2 and in sustaining Akt survival signal. J. Cell. Physiol..

[40] Williams KC, Coppolino MG (2011). Phosphorylation of membrane type 1-matrix metalloproteinase (MT1-MMP) and its vesicle-associated membrane protein 7 (VAMP7)-dependent trafficking facilitate cell invasion and migration. J. Biol. Chem..

[41] Steffan JJ (2014). Supporting a role for the GTPase Rab7 in prostate cancer progression. PLoS ONE.

[42] Gutierrez MG, Munafo DB, Beron W, Colombo MI (2004). Rab7 is required for the normal progression of the autophagic pathway in mammalian cells. J. Cell Sci..

[43] Jager S (2004). Role for Rab7 in maturation of late autophagic vacuoles. J. Cell Sci..

[44] Su H, Li F, Ranek MJ, Wei N, Wang X (2011). COP9 signalosome regulates autophagosome maturation. Circulation.

[45] Chen W, Lu C, Hong J (2018). TRIM15 exerts anti-tumor effects through suppressing cancer cell invasion in gastric adenocarcinoma. Med. Sci. Monit..

[46] Ma Y (2016). Downregulation of TRIM27 expression inhibits the proliferation of ovarian cancer cells in vitro and in vivo. Lab. Invest..

[47] Liang Q (2019). TRIM47 is up-regulated in colorectal cancer, promoting ubiquitination and degradation of SMAD4. J. Exp. Clin. Cancer Res..

[48] Geng B (2019). An TRIM59-CDK6 axis regulates growth and metastasis of lung cancer. J. Cell Mol. Med..

[49] Sun Y (2017). TRIM59 facilitates the proliferation of colorectal cancer and promotes metastasis via the PI3K/AKT pathway. Oncol. Rep..

[50] Liu M (2018). Downregulation of TRIM58 expression is associated with a poor patient outcome and enhances colorectal cancer cell invasion. Oncol. Rep..

[51] Mandell MA (2014). TRIM proteins regulate autophagy and can target autophagic substrates by direct recognition. Dev. Cell.

[52] Jena, K. K. et al. TRIM16 controls assembly and degradation of protein aggregates by modulating the p62-NRF2 axis and autophagy. *EMBO J.* **37**, e98358 (2018). 10.15252/embj.201798358 PMC6138442 30143514

[53] Kinsey C (2014). Plac8 links oncogenic mutations to regulation of autophagy and is critical to pancreatic cancer progression. Cell Rep..

[54] Müller MP, Goody RS (2018). Molecular control of Rab activity by GEFs, GAPs and GDI. Small GTPases.

[55] Varshavsky A (2017). The ubiquitin system, autophagy, and regulated protein degradation. Annu. Rev. Biochem..

[56] Song P, Trajkovic K, Tsunemi T, Krainc D (2016). Parkin modulates endosomal organization and function of the endo-lysosomal pathway. J. Neurosci..

[57] Margiotta A, Progida C, Bakke O, Bucci C (2017). Rab7a regulates cell migration through Rac1 and vimentin. Biochim. Biophys. Acta Mol. Cell Res..

[58] Wang W (2017). Internalized CD44s splice isoform attenuates EGFR degradation by targeting Rab7A. Proc. Natl Acad. Sci. USA.

