# Supplementary material for: TRIM39 deficiency inhibits tumor progression and autophagic flux in colorectal cancer via suppressing the activity of Rab7
Source: Cell Death Dis. 2021 Apr 12;12(4):391. doi: 10.1038/s41419-021-03670-3 (PMC8041807; doi:10.1038/s41419-021-03670-3)
Supplement: Supplementary file 1 — suppl. info. [file 41419_2021_3670_MOESM1_ESM.docx]

Supplementary Materials

**Table S1.** Primer sequence for synthesizing shRNA and siRNAs

| Name |  | Primer sequence |
| --- | --- | --- |
| shTRIM39-1# |  | F:CCGGGTATGCTTGATATGTGCAATTCTCGAGAATTGCACATATCAAGCATACTTTTTG |
|  |  | R:AATTCAAAAAGTATGCTTGATATGTGCAATTCTCGAGAATTGCACATATCAAGCATAC |
| shTRIM39-2# |  | F:CCGGCGATGCTACACAGGAGTACAACTCGAGTTGTACTCCTGTGTAGCATCGTTTTTG |
|  |  | R:AATTCAAAAACGATGCTACACAGGAGTACAACTCGAGTTGTACTCCTGTGTAGCATCG |
| shRab7 |  | F:CCGGGGCTAGTCACAATGCAGATATCTCGAGATATCTGCATTGTGACTAGCCTTTTTG |
|  |  | R:AATTCAAAAAGGCTAGTCACAATGCAGATATCTCGAGATATCTGCATTGTGACTAGCC |
| shp53 |  | F:CCGGCGGCGCACAGAGGAAGAGAATCTCGAGATTCTCTTCCTCTGTGCGCCGTTTTTG |
|  |  | R:AATTCAAAAACGGCGCACAGAGGAAGAGAATCTCGAGATTCTCTTCCTCTGTGCGCCG |
| siTRIM39-1 |  | GCAGAUCUUGAGGGAGUUUTT |
| siTRIM39-2 |  | GGCAACUAGGCAGUAUGGUGGAAAU |
| siTRIM39-3 |  | CCAAGGCGUUUCACCUUCUACCCUU |

**Table S2.** Primer sequence for RT-PCR

| Gene | semi-RT-PCR Primer sequence (5’ to 3’) |
| --- | --- |
| GAPDH | F:GTGGATATTGTTGCCATCAA |
|  | R:ATTCGTTGTCATACCAGGAA |
| TRIM39 | F:GGCAAGTGCTTACAGTCA |
|  | R:TCAGGGAGTGGAGTCAAC |


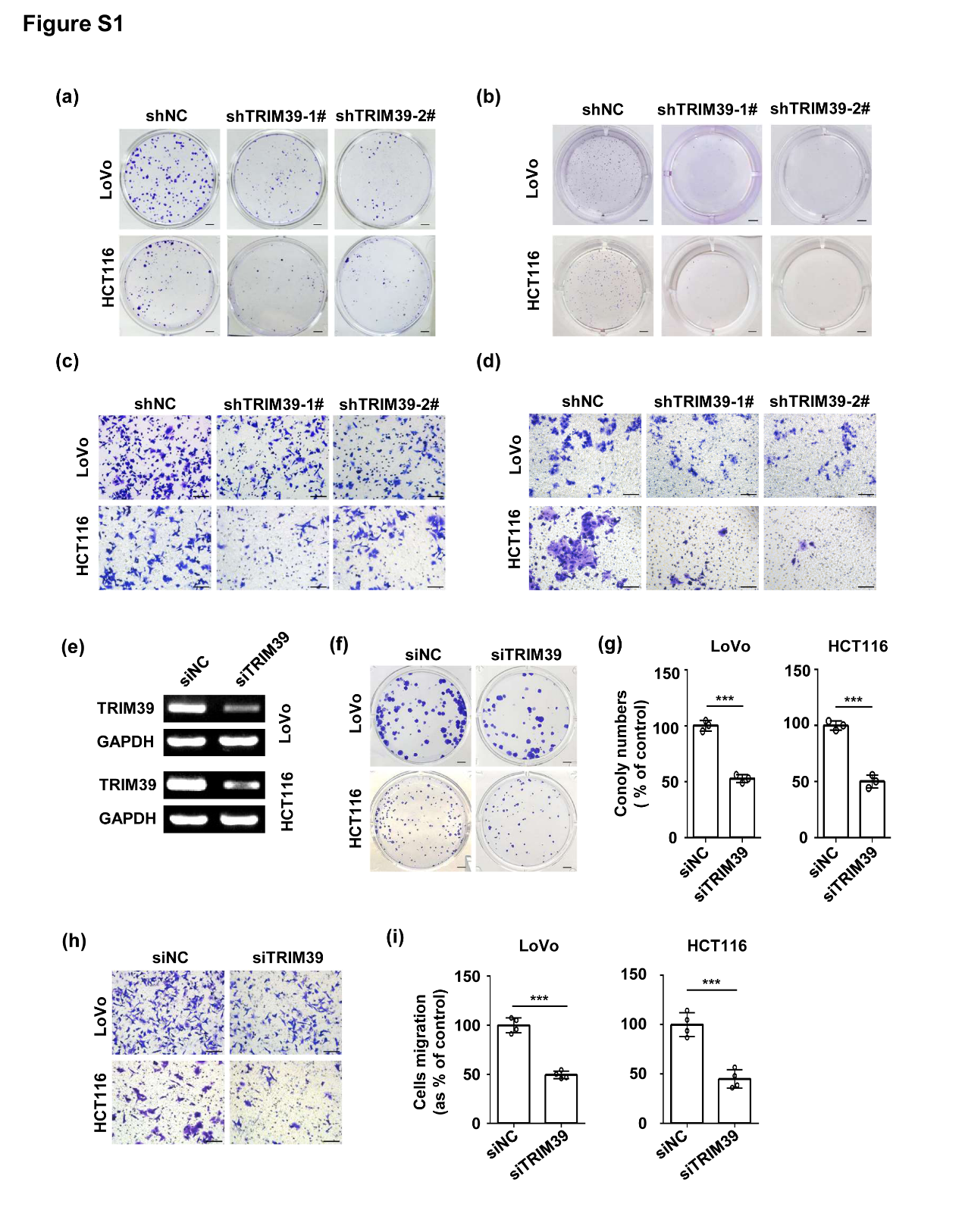


**Figure S1. a-b** The representative images of colony formation assay (**a**) and soft-agar colony formation assay (**b**) in Fig. 1f, 1g. Scale bar, 2 mm. **c-d** The representative images of migration assay (**c**) and invasion assay (**d**) in Fig. 1h, 1i. Scale bar, 50 μm. **e** RT-PCR analysis of TRIM39 knockdown efficiency in LoVo and HCT116 cells transfected with pooled TRIM39 siRNAs for 48 h. **f** The representative images of colony formation assay of LoVo and HCT116 cells transfected with pooled TRIM39 siRNAs. Scale bar, 2 mm. **g** Quantification of colony formation assay in (**f**). Data shown as mean ± SD. n = 3 samples per group. Student’s *t* test. ***, *P* < 0.001. **h** The representative images of migration assay of LoVo and HCT116 cells transfected with pooled TRIM39 siRNAs. Scale bar, 50 μm. **i** Quantification of migration assay in (**h**). Data shown as mean ± SD. n = 3 samples per group, four fields per sample. Student’s *t* test. ***, *P* < 0.001.


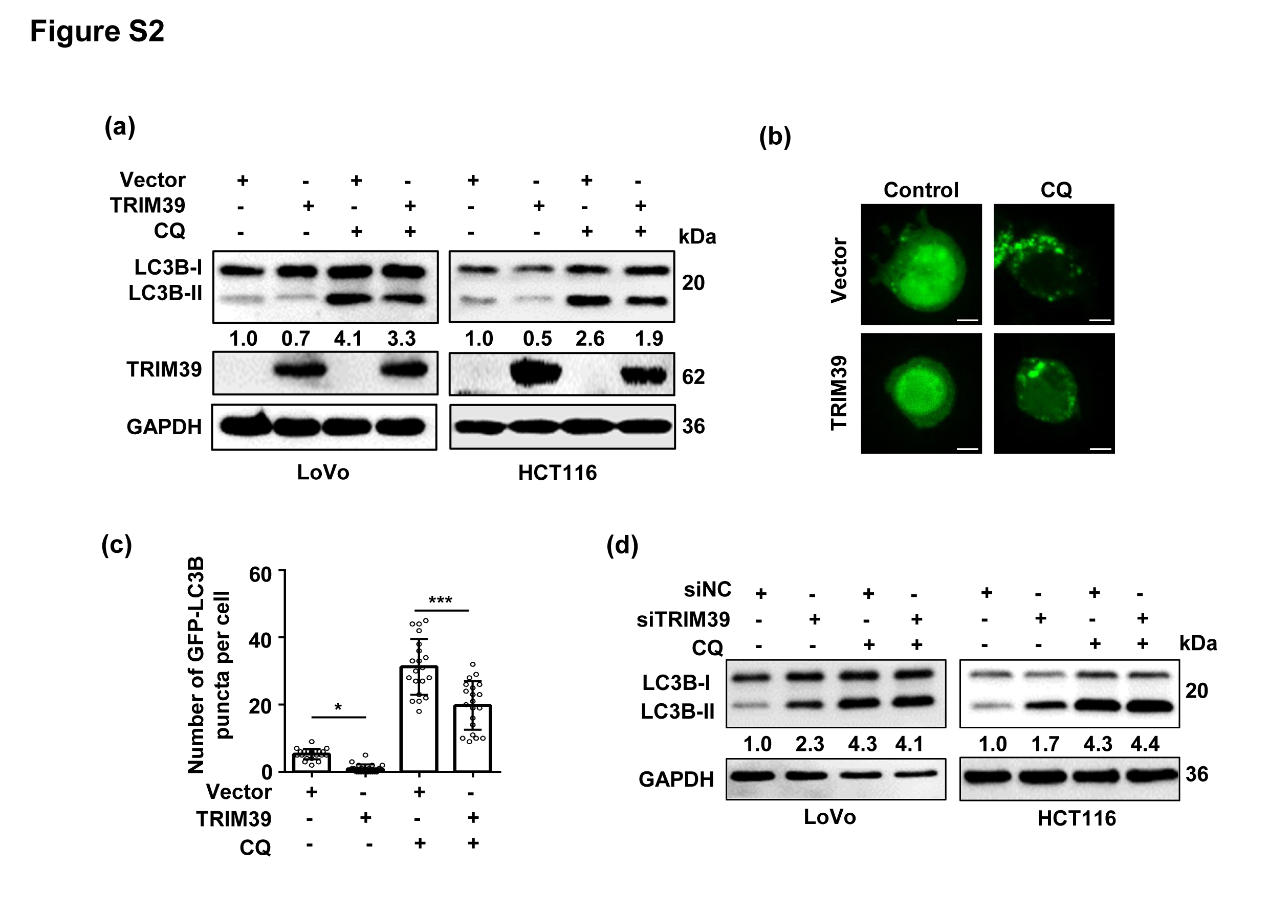


**Figure S2.** **a** LoVo and HCT116 cells were transfected with Vector or FLAG-TRIM39 for 48 h and treated with or without CQ (50 nM) for the last 4 h. The levels of LC3B-II were detected by western blot. **b** LoVo cells were co-transfected with GFP-LC3B and empty-vector or FLAG-TRIM39 and then treated with or without CQ (50 nM) for the last 4 h. Representative confocal microscopy images of GFP-LC3B puncta were shown. Scale bar, 5μm. **c** Quantification of GFP-LC3B puncta per cell treated as in (**b**). Data shown as mean ± SD. 20 cells were scored. One-way ANOVA. *, P < 0.05; ***, P < 0.001. **d** Western blot analysis of LC3-II levels in LoVo and HCT116 cells transfected with pooled TRIM39 siRNAs.


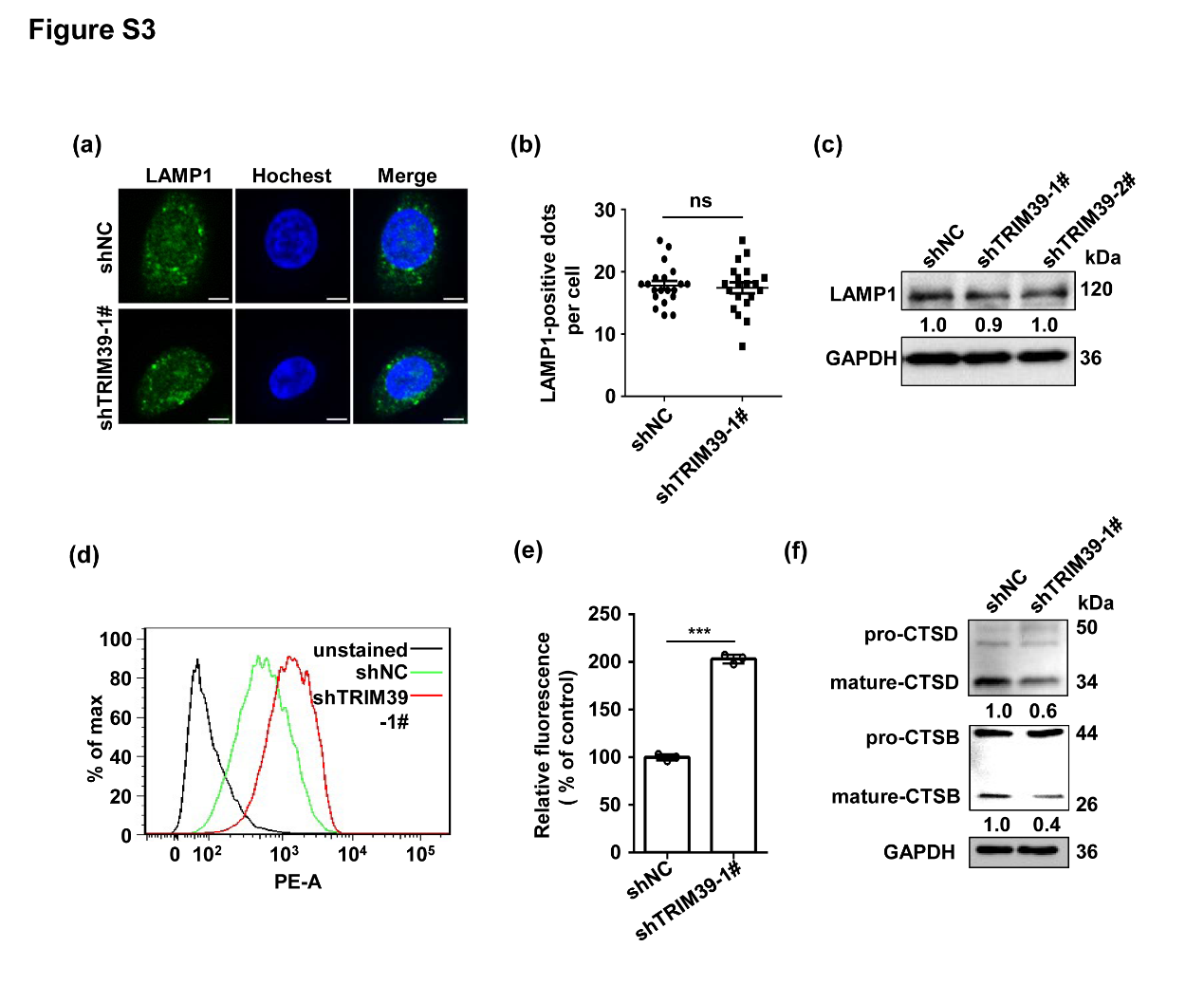


**Figure S3. a** LoVo cells with TRIM39 stably knockdown were fixed and immunofluorescently stained for LAMP1. Representative confocal microscopy images were shown. Scale bar, 5 μm. **b** Quantification of LAMP1-positive dots per cell treated as in (**a**). Data shown as mean ± SD. 20 cells were scored. Student’s *t* test. ns, no significance. **c** Western blot analysis of LAMP1 levels in TRIM39 stably knockdown HCT116 cells. **d** TRIM39 stably knockdown HCT116 cells were stained with LysoTracker red at 37°C for 30 min. The fluorescent intensity of stained cells was detected by flow cytometer. **e** Quantification of relative fluorescence in (**d**). Data shown as mean ± SD. Student’s *t* test. ***, *P* < 0.001. **f** Western blot analysis of CTSB, CTSD levels in TRIM39 stably knockdown HCT116 cells.


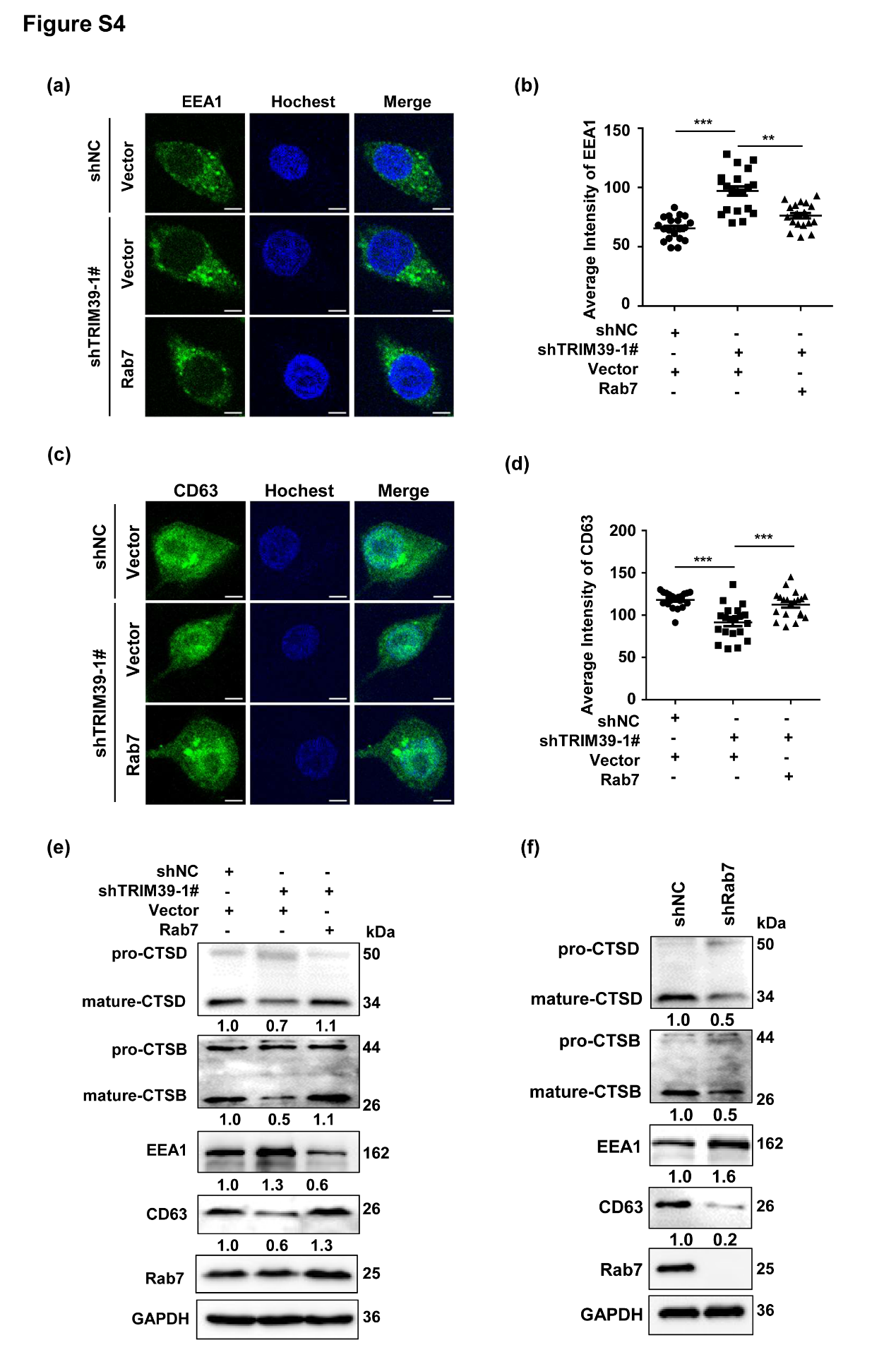


**Figure S4. a** TRIM39 stably knockdown HCT116 cells were infected with Empty vector and Rab7 overexpression lentivirus. Cells were then fixed and immunofluorescently stained for EEA1. Representative confocal microscopy images were shown. Scale bar, 5 μm. **b** The fluorescence intensity of EEA1 in (**a**) was calculated. Data shown as mean ± SD. 20 cells were scored. One-way ANOVA. **, P < 0.01; ***, P < 0.001. **c** TRIM39 stably knockdown HCT116 cells were infected with Empty vector and Rab7 overexpression lentivirus. Cells were then fixed and immunofluorescently stained for CD63. Representative confocal microscopy images were shown. Scale bar, 5 μm. **d** The fluorescence intensity of CD63 in (**c**) was calculated. Data shown as mean ± SD. 20 cells were scored. One-way ANOVA. ***, P < 0.001. **e** TRIM39 stably knockdown HCT116 cells were infected with Empty vector and Rab7 overexpression lentivirus. The protein levels of CTSB, CTSD, EEA1 and CD63 were detected by western blot. **f** Western blot assay of CTSB, CTSD, EEA1 and CD63 levels in HCT116 cells with Rab7 stably knockdown.


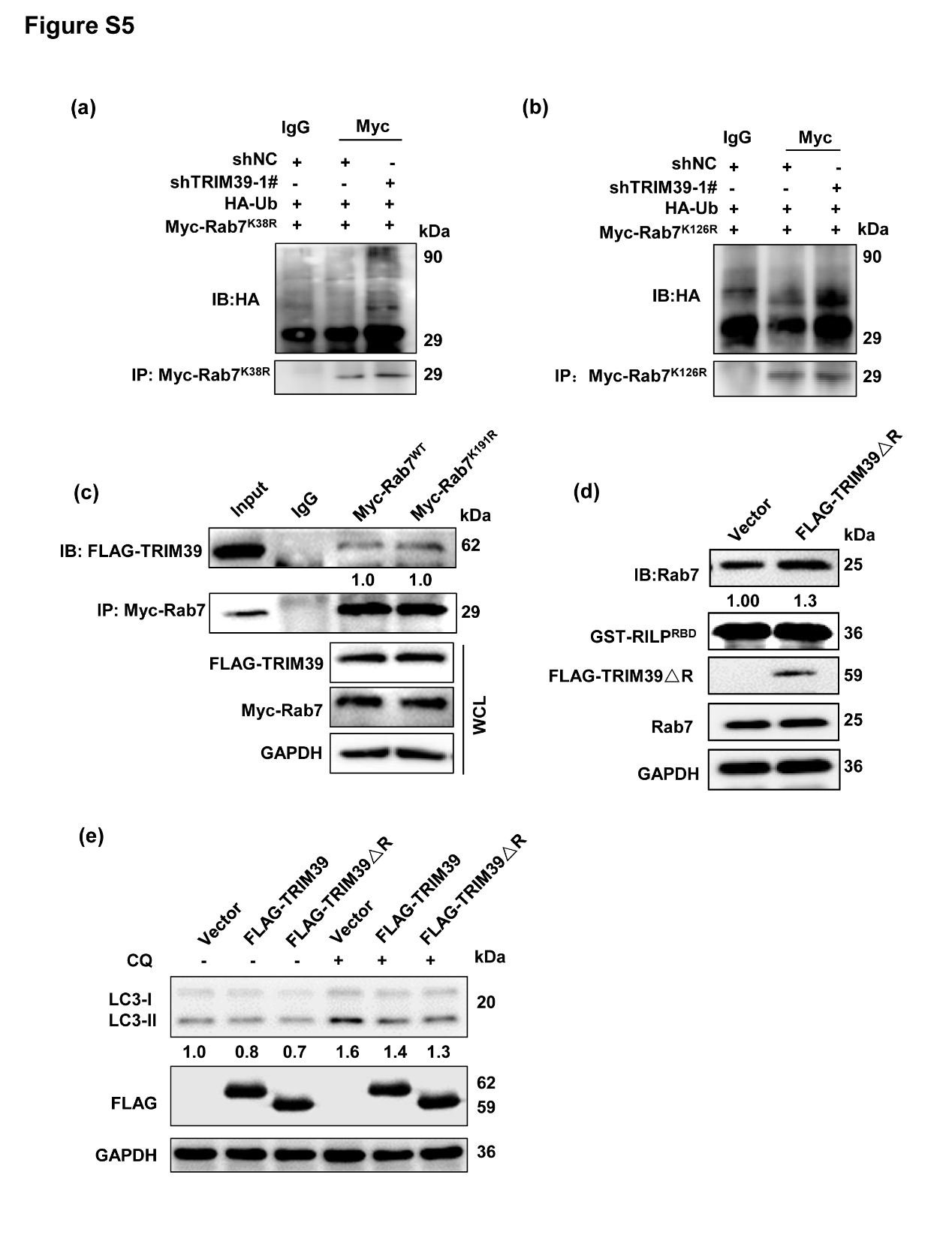


**Figure S5. a** Myc-Rab7^K38R^ was co-transfected with HA-ubiquitin into shNC or TRIM39 stably knockdown HCT116 cells for 48 h. Cell lysates were immunoprecipitated with anti-Myc antibody and immunoblotted with anti-HA and anti-Myc antibody. **b** Myc-Rab7^K126R^ was co-transfected with HA-ubiquitin into shNC or TRIM39 stably knockdown HCT116 cells for 48 h. Cell lysates were immunoprecipitated with anti-Myc antibody and immunoblotted with anti-HA and anti-Myc antibody. **c** HEK293T cells were transfected with FLAG-TRIM39 and Myc-Rab7^WT^ or Myc-Rab7^K191R^ for 48 h. Total cell lysates were subjected to immunoprecipitation with anti-Myc antibody. Then the precipitates were examined with anti-FLAG. **d** GST-RILP^RBD^ fusion protein immobilized on Glutathione-Sepharose beads were incubated with Vector or FLAG-TRIM39ΔR overexpressed LoVo cell lysates at 4°C for 4 h. Rab7 and GST were detected in the washed beads by western blot. **e** HCT116 cells were transfected with Vector, FLAG-TRIM39 or FLAG-TRIM39△R for 48 h and treated with or without CQ (50 nM) for the last 4 h. The levels of LC3B-II were detected by western blot.


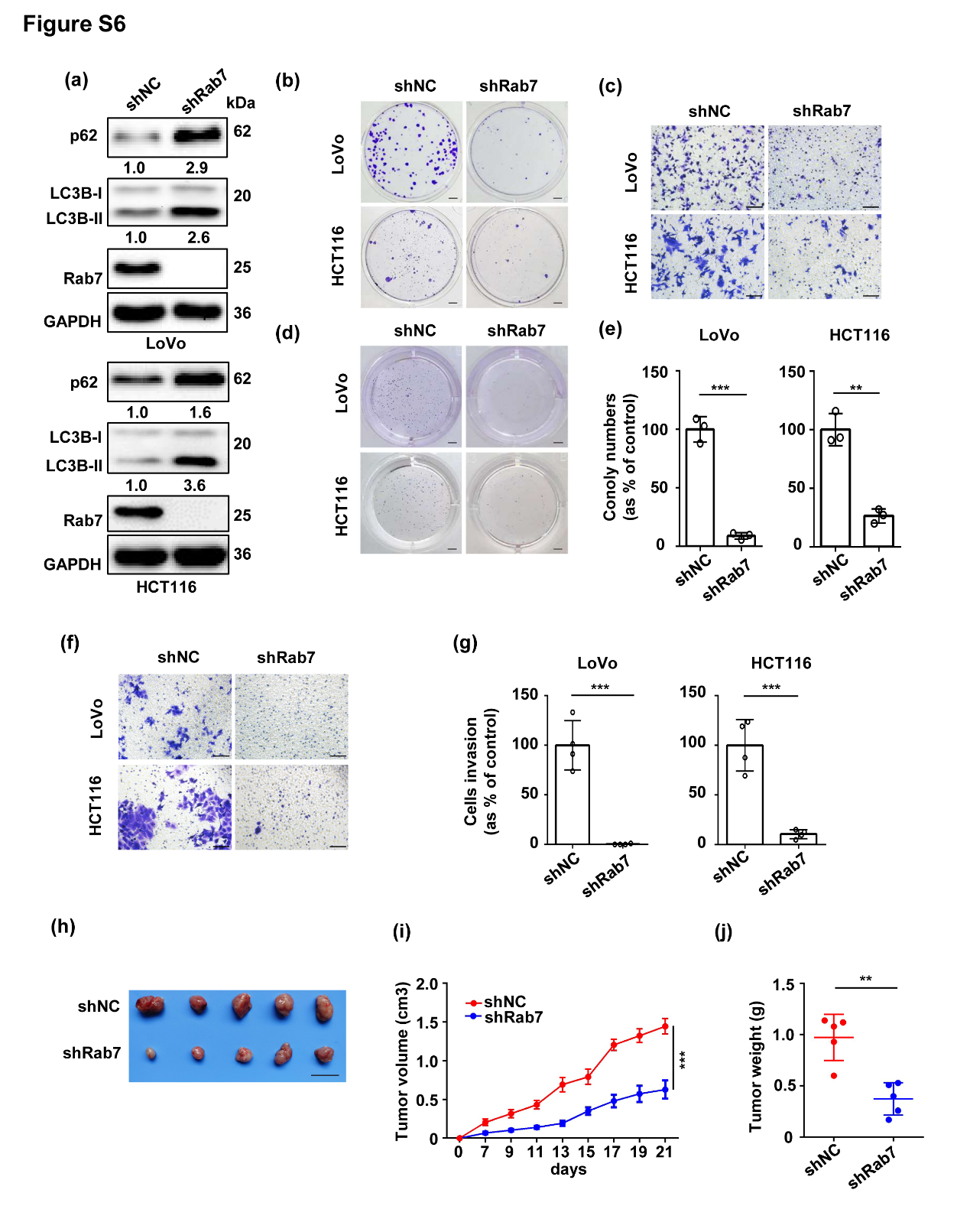


**Figure S6. a** Western blot assay of LC3B-II and p62 levels in LoVo and HCT116 cells with Rab7 stably knockdown. **b** The representative images of colony formation assay in Fig. 6c. Scale bar, 2 mm. **c** The representative images of migration assay in Fig. 6e. Scale bar, 50 μm. **d** The representative images of soft-agar colony formation assay of LoVo and HCT116 cells with Rab7 stably knockdown. Scale bar, 2 mm. **e** Quantification of soft-agar colony formation assay in (**d**). Data shown as mean ± SD. n = 3 samples per group. Student’s *t* test. **, *P* < 0.01; ***, *P* < 0.001. **f** The representative images of invasion assay of LoVo and HCT116 cells with Rab7 stably knockdown. Scale bar, 50 μm. **g** Quantification of invasion assay in (**f**). Data shown as mean ± SD. n = 3 samples per group, four fields per sample. Student’s *t* test. ***, *P* < 0.001. **h-j** Rab7 stably knockdown HCT116 cells or control cells (shNC) were subcutaneously injected into nude mice. Representative images of the xenograft tumors (**h**), tumor growth curves (**i**) and tumor weight (**j**) were shown. n = 5 per group; Data shown as mean ± SD for tumor weight and mean ± SEM for tumor growth. Scale bar, 2 cm. Student’s *t* test. **, *P* < 0.01; ***, *P* < 0.001.


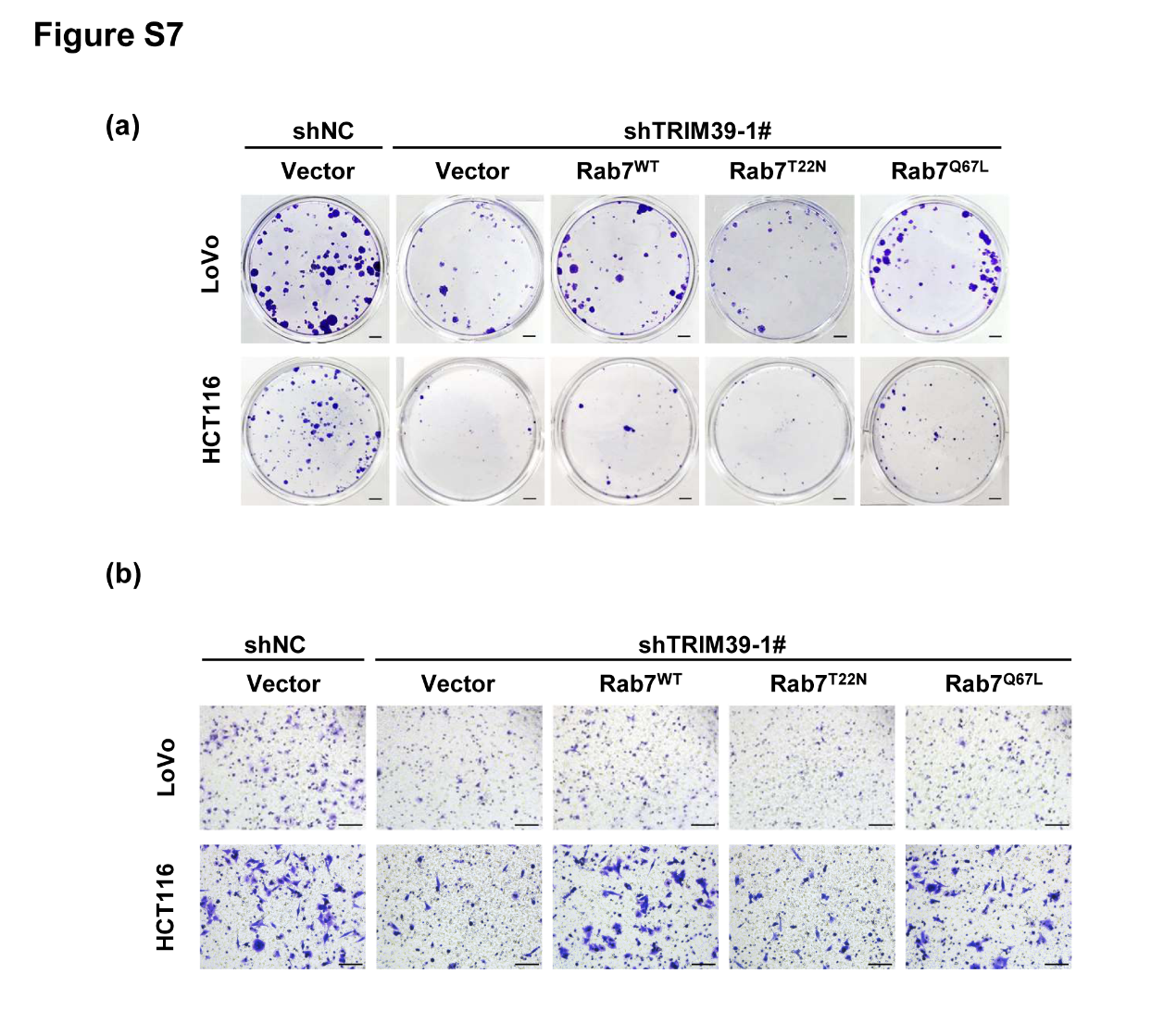


**Figure S7. a** Representative images of colony formation assay in Fig. 6d. Scale bar, 2 mm. **b** Representative images of migration assay in Fig. 6f. Scale bar, 50 μm.


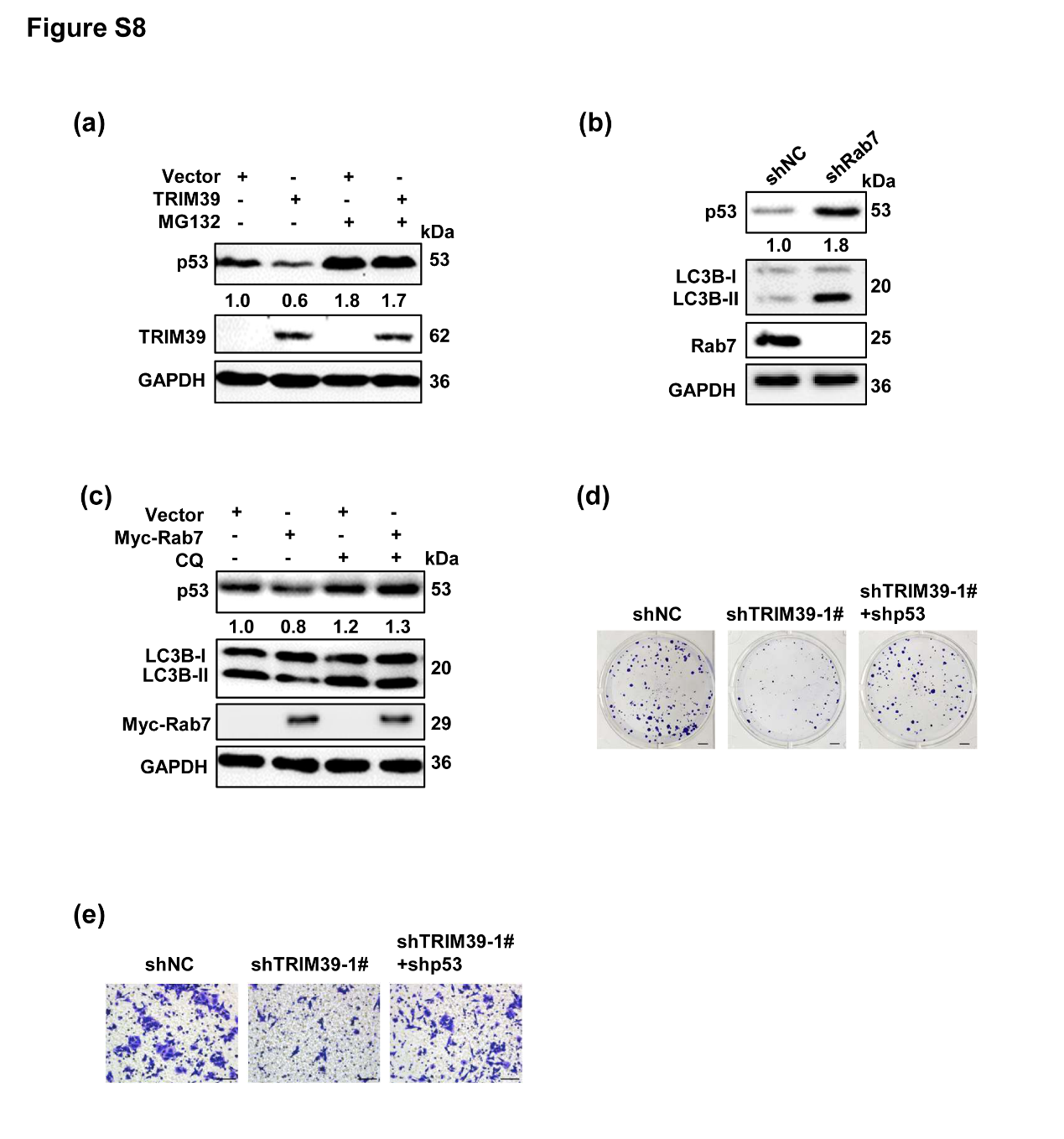


Figure S8. a HCT116 cells were transfected with empty vector or FLAG-TRIM39 for 48 h and then treated with or without MG132 (10 μM) for the last 12 h. The levels of p53 were detected by western blot. b Western blot assay of p53 levels in Rab7 stably knockdown HCT116 cells. c HCT116 cells were transfected with Vector or Rab7 for 48 h and treated with or without CQ (50 nM) for the last 4 h. The levels of p53 were detected by western blot. d Representative images of colony formation assay in Fig. 8k. Scale bar, 2 mm. e Representative images of migration assay in Fig. 8l. Scale bar, 50 μm.
